# Association of changes in expression of *HDAC* and *SIRT* genes after drug treatment with cancer cell line sensitivity to kinase inhibitors

**DOI:** 10.1080/15592294.2024.2309824

**Published:** 2024-02-18

**Authors:** Julia Krushkal, Yingdong Zhao, Kyle Roney, Weimin Zhu, Alan Brooks, Deborah Wilsker, Ralph E. Parchment, Lisa M. McShane, James H. Doroshow

**Affiliations:** a Biometric Research Program, Division of Cancer Treatment and Diagnosis, National Cancer Institute, Rockville, MD, USA; b Department of Biostatistics and Bioinformatics, George Washington University, Washington, DC, USA; c Clinical Pharmacodynamic Biomarkers Program, Applied/Developmental Research Directorate, Frederick National Laboratory for Cancer Research, Frederick, MD, USA; d Division of Cancer Treatment and Diagnosis and Center for Cancer Research, National Cancer Institute, Bethesda, MD, USA

**Keywords:** Histone deacetylase, sirtuin, dasatinib, chemosensitivity, HDAC5

## Abstract

Histone deacetylases (HDACs) and sirtuins (SIRTs) are important epigenetic regulators of cancer pathways. There is a limited understanding of how transcriptional regulation of their genes is affected by chemotherapeutic agents, and how such transcriptional changes affect tumour sensitivity to drug treatment. We investigated the concerted transcriptional response of *HDAC* and *SIRT* genes to 15 approved antitumor agents in the NCI-60 cancer cell line panel. Antitumor agents with diverse mechanisms of action induced upregulation or downregulation of multiple *HDAC* and *SIRT* genes. *HDAC5* was upregulated by dasatinib and erlotinib in the majority of the cell lines. Tumour cell line sensitivity to kinase inhibitors was associated with upregulation of *HDAC5, HDAC1*, and several *SIRT* genes. We confirmed changes in *HDAC* and *SIRT* expression in independent datasets. We also experimentally validated the upregulation of HDAC5 mRNA and protein expression by dasatinib in the highly sensitive IGROV1 cell line. HDAC5 was not upregulated in the UACC-257 cell line resistant to dasatinib. The effects of cancer drug treatment on expression of *HDAC* and *SIRT* genes may influence chemosensitivity and may need to be considered during chemotherapy.

## Introduction

The products of the histone deacetylase (*HDAC*) and sirtuin (*SIRT*) gene families deacetylate functional lysine groups in histones and non-histone proteins [1,2]. Deacetylation of protein acetyl lysine substrates by the ‘classical’ HDAC proteins (class I, II, and IV HDACs) and by the SIRT proteins (SIRTs, or class III HDACs) is an integral part of epigenetic regulation of numerous important cellular and developmental processes [1–6]. HDACs and SIRTs participate in epigenetic regulation of diverse biological processes including, e.g., chromatin regulation, transcription, cell cycle progression, metabolic processes, inflammation and immune response, cell survival, and other important cellular and organismal pathways [1–3,5–14]. In addition to deacetylation of histones and non-histone proteins, some HDACs are also involved in other molecular mechanisms of epigenetic regulation. For example, HDAC1 directly affects DNA methylation by interacting with DNA methyltransferase 1 (DNMT1) and via participation in a molecular complex which controls DNMT1 abundance [15–17]. SIRTs, in addition to their roles in acetyl lysine deacetylation, also function as mono-ADP-ribosyltransferases and have other catalytic activities [1,9].

Biological activity of many HDACs and SIRTs is altered in cancer. These changes play an important role in epigenetic deregulation within and outside tumour cells via the induction of transcriptional changes and direct modification of multiple tumour suppressor genes, oncogenes, and genes involved in diverse cellular processes, and through modulation of the inflammatory and immune pathways in the tumour microenvironment [1–4,6,8,13,17,18]. HDACs and SIRTs are important targets for cancer therapy through inhibition of their enzymatic activity by the HDAC and sirtuin inhibitors, which leads to a broad modulation of downstream biological pathways regulated by these proteins [1–3,18–21].

In addition to the changes that occur in the catalytic activity of HDACs and SIRTs in cancer cells, mRNA and protein expression of many HDACs and a number of SIRTs is also dysregulated in tumours [2,3,9,10,13,18,22–25]. Both upregulation and downregulation of HDAC and SIRT levels in tumours have been reported, suggesting a context-dependent effect of such changes [2,3,8,10,13,22–28]. HDAC proteins are frequently overexpressed in many tumour categories [2,3,22–25]. However, direct oncogenic effects of their upregulation have been debated, and in specific tumour types some HDACs lose their expression or function, which may also promote tumorigenesis [3,8,25–28]. For example, both overexpression and downregulation or loss of HDAC2 and HDAC5 have been reported in tumours, and both types of changes were associated with negative outcomes [8,26–28]. While the direct molecular impact of expression changes of HDACs in cancer remains an open question, mRNA and protein overexpression of nearly all HDACs in specific tumour categories has been associated with inferior clinical outcomes and with promotion of cancer cell proliferation and aggressiveness [18,22–25].

Some studies suggested a direct benefit of downregulating or depleting individual HDACs and SIRTs for improving outcomes of chemotherapy. For example, HDAC5 depletion in MCF-7 and HeLa cells using *HDAC5* siRNA improved tumour sensitivity to the DNA damaging agents doxorubicin and cisplatin [8]. Similarly, overexpression of *HDAC1* was observed in multidrug resistant neuroblastoma cell lines and increased resistance of melanoma cells to sodium butyrate, whereas *HDAC1* siRNA knockdown sensitized neuroblastoma cells to etoposide [2,29,30]. *SIRT2* knockdown in the melanoma cell lines MDA-MB-435S and WM853 sensitized them to dasatinib [31]. Depletion of *SIRT1* in the AML cell line K-562 increased sensitivity to HSP90 inhibitors [14]. In contrast, increased mRNA and protein expression of SIRT3 and SIRT4 has been found to be beneficial for patient outcomes in hepatocellular carcinoma (HCC), and higher levels of SIRT3 mRNA and protein expression in HCC cell lines increased sensitivity to cisplatin, doxorubicin, epirubicin, sorafenib, and regorafenib [32]. Even though the effects of *SIRT3* may be specific to the types of tumours, upregulation of both *SIRT3* and *SIRT4* is currently being pursued as a potential strategy to improve cancer treatment outcomes [32].

Separate from direct enzymatic targeting of HDACs and SIRTs in cancer therapy, cancer drugs may also affect their mRNA or protein levels. Chemotherapy treatment of malignant cells with antitumor agents induces a broad range of transcriptional changes in a variety of genes [21,33–37]. Despite evidence for widespread transcriptome changes in tumours after drug treatment, detailed and systematic knowledge of the direction and amplitude of transcriptional changes of individual *HDAC* and *SIRT* genes in response to various agents remains limited. Our understanding of downstream effects of transcriptional upregulation or downregulation of *HDAC* and *SIRT* genes in response to cancer drug treatment is also limited. Since both histone deacetylating families represent important targets in cancer therapy, perturbations of their expression may have unforeseen effects on drug sensitivity or resistance. It is important to evaluate both the influence of chemotherapy on HDAC and SIRT expression and the potential effect of possible changes in their expression on drug response. To address this important question, we analysed data from the well-characterized NCI-60 cancer cell line panel to identify concerted transcriptional changes in *HDAC* and *SIRT* genes after treatment with 15 approved antitumor agents. We also examined the association of post-treatment changes and of baseline levels of *HDAC* and *SIRT* genes with tumour cell line response to treatment with each agent.

## Materials and methods

The overall workflow of the study and the sources of data are provided in Figure S1. The initial discovery analysis utilized the publicly available NCI-TPW (the NCI Transcriptional Pharmacodynamics Workbench) dataset generated by the US National Cancer Institute (NCI). This computational analysis identified *HDAC* and *SIRT* genes with concerted expression changes and also identified those genes whose expression changes or baseline expression were associated with drug response. mRNA and protein expression changes were validated experimentally using RT-PCR and Western blots by our group and also computationally using publicly available data from NCBI GEO (National Center for Biotechnology Information Gene Expression Omnibus) and biomedical publications. We used publicly available data from NCI-TPW and NCBI GEO to perform additional computational analyses of miRNAs involved in the regulation of HDAC5, which emerged as the top lead in the discovery part of the analysis.

### Drug response data

Expression data and drug response measures were obtained from NCI-TPW, which contains drug response and transcriptional data for the NCI-60 cancer cell line panel treated with 15 FDA-approved antitumor agents (https://tpwb.nci.nih.gov) [33]. Cell line drug sensitivity was analysed using log_10_(GI50) values, where GI50 is a drug concentration (μM) producing 50% growth inhibition [38]. The NCI-TPW GI50 values were generated for each antitumor drug after a 48 hr exposure to the agent according to the NCI-60 drug screening protocols [33]. The response measures were available for low (clinically achievable) and high (in-vitro active) concentrations of each agent. The NCI-TPW dataset included the broad-spectrum HDAC inhibitor vorinostat (suberoylanilide hydroxamic acid, or SAHA) [2] at 1 and 5 µM/L for the low and high concentrations, respectively. Additional antitumor agents included the epigenetic DNMT1 inhibitor azacytidine (1 and 5 µM), the kinase inhibitors dasatinib (0.1 and 2 µM), erlotinib (1 and 10 µM), sunitinib (0.2 and 2 µM), lapatinib (1 and 10 µM) and sorafenib (5 and 10 µM), the proteasome inhibitor bortezomib (0.01 and 0.1 µM), the mTOR inhibitor sirolimus (0.01 and 0.1 µM), the Hsp90 inhibitor geldanamycin (0.1 and 1 µM), the DNA damaging agents doxorubicin (0.1 and 1 µM), gemcitabine (0.2 and 2 µM), cisplatin (3 and 15 µM), and topotecan (0.01 and 1 µM), and the microtubule stabilizing agent paclitaxel (0.01 and 0.1 µM) [33].

### HDAC and SIRT gene expression measures

We analysed gene expression levels in untreated cell lines and expression changes after treatment for all *HDAC* and *SIRT* genes which had the data in the NCI-TPW dataset. They included 9 *HDAC* genes (*HDAC1, HDAC2, HDAC3, HDAC4, HDAC5, HDAC6, HDAC7, HDAC9*, and *HDAC11*) and all 7 *SIRT* genes (*SIRT1, SIRT2, SIRT3, SIRT4, SIRT5, SIRT6*, and *SIRT7*). *HDAC8* and *HDAC10* were not analysed, as their expression was not profiled in the NCI-TPW dataset. Both expression data in cell lines treated with vehicle only (to which we refer as baseline expression) and expression measures after treatment were analysed. Quality control, RNA normalization, data processing, and validity of expression measures for the NCI-TPW dataset were described previously [33–35]. In brief, expression data were obtained from time course Affymetrix HG-U133A microarray experiments [33]. The gene expression measures at 2, 6, and 24 hr after treatment of the 60 cell lines with low and high concentrations of each agent were compared to time-matched baseline control measures, collected from cells treated with vehicle only [33]. The measurements for the microarray probe sets in the NCI-TPW dataset were processed using background subtraction and RMA array normalization [39]. The log_2_ expression values from different probe sets within a single gene from a single microarray were combined and averaged. We refer to these expression changes as log_2_FC, i.e., log_2_ of the post-treatment fold change of expression of each gene in each cell line. The log_2_FC values provide the difference between the log_2_ of the averaged gene expression value in a cell line treated with an agent and the log_2_ of the averaged expression value of that gene in the same cell line that was treated only with vehicle and collected at time-matched intervals at 2, 6, or 24 hr after treatment. Post-treatment expression changes of selected genes were previously validated in a replicate study using a Fluidigm BioMark™ qRT-PCR System [33].

### Analysis of baseline gene expression and concerted transcriptional changes of HDAC and SIRT genes after drug treatment

To examine the direction of expression changes of the *HDAC* and *SIRT* genes after treatment with each of the 15 agents, we used our previously described definition of concerted transcriptional changes based on the threshold of ≤ 15 cell lines with discordant direction of transcriptional change at any given time point and concentration [34,35]. We considered a gene to have concerted upregulation or concerted downregulation for those microarray experiments (specific to an antitumor agent, concentration, and post-treatment time), in which the expression of the vast majority of the cell lines changed in the same direction, with no more than 15 cell lines showing an expression change in the opposite direction. Our earlier study of five agents in the NCI-TPW dataset [34] showed that this threshold provided an appropriate measure of concerted transcriptional changes and was concordant with independent resources, and that the probability of ≤ 15 cell lines to be expressed in the opposite direction at random is low, between 6.96 × 10^−5^ and 1.54 × 10^−3 34^. For those genes and conditions which satisfied the criteria for concerted expression changes, we refer to the direction of the changes (upregulation or downregulation) in the majority of the cell lines after treatment as consensus changes.

Baseline expression for each *HDAC* and *SIRT* gene in a given cell line was computed as a median of log_2_-transformed expression values in the matched untreated control cell lines for all 15 agents at 6 hr after the start of the experiment. We had previously selected the 6 hr time point as providing the most biologically relevant representation of baseline gene expression in the NCI-TPW data. NCI-TPW baseline gene expression measures at 6 hr were consistent with similar measures at 2 and 24 hr [33–35].

### Analysis of correlation of cancer drug response with baseline HDAC and SIRT gene expression and expression changes after treatment

For each low and high concentration of each agent and for each time point (2, 6, and 24 hr), we examined Spearman and Pearson correlation of drug response (log(GI50)) of the NCI-60 cell lines with the amplitude of gene expression changes (log_2_FC). We also examined Spearman and Pearson correlation of log(GI50) of each agent with baseline gene expression in the untreated NCI-60 control cell lines. Significance of the correlations was evaluated using the Benjamini-Hochberg adjustment for false discovery rate (FDR) [40]. The FDR adjustment was performed separately for Spearman and Pearson correlation *p*-values. For each type of correlation (Spearman or Pearson), the FDR adjustment was applied to a single set of *p-*values from all time points and both concentrations, while also including the *p-*values from baseline correlation analysis. Here and below, we refer to the *p-*values prior to FDR adjustment as *p*_0_, and FDR adjusted *p-*values as *p*_FDR_. Correlation analyses were performed using R v. 3.5.3 and 4.2.1 and Microsoft Excel. Graphical output for NCI-TPW data was generated using the graphical interfaces of NCI-TPW [33] and TPWShiny (available for download at https://brb.nci.nih.gov/TPWshiny/) [41].

### Validation of expression changes in HDAC and SIRT genes and protein products after drug treatment using publicly available independent datasets

In order to confirm the direction of concerted changes in *HDAC* and *SIRT* genes in response to dasatinib and vorinostat observed in the NCI-TPW data, we examined independent mRNA and protein expression datasets. We analysed publicly available datasets from the NCBI GEO [https://www.ncbi.nlm.nih.gov/geo/] or described in biomedical publications [7,42,43]. We extracted pre-treatment and post-treatment gene and isoform mRNA expression data for *HDACs* and *SIRTs* from the NCBI GEO datasets GSE51083 [44] and GSE69395 [45] for cell lines treated with dasatinib, and mRNA expression data from GSE84205 [46] and GSE43010 [47] for cell lines treated with vorinostat. These datasets provided Affymetrix and Illumina gene expression microarray data prior to drug treatment and after treatment at various time points. Depending on whether transcript isoform data or gene level data were available in the annotation of each dataset, the direction of transcriptional changes of each *HDAC* and *SIRT* gene in each cell line was summarized using the log_2_ of transcriptional changes for transcript isoforms and/or entire genes, and averaged among the probe sets. For those datasets where technical replicates were available, the log_2_-transformed expression changes were averaged among the technical replicates.

The NCBI GEO dataset GSE51083 included Illumina HT-1 microarray expression data (multiple transcripts per gene) for the chronic myelogenous leukaemia (CML) K-562 cell line, profiled at baseline and at 4, 8, and 24 hr after treatment with 100 nM of dasatinib [44]. K-562 is a part of the NCI-60 panel, and the 100 nM dasatinib concentration used in GSE51083 was also used as the low concentration of dasatinib in NCI-TPW. For each gene, we used the 24 hr time point, which was profiled in both datasets, to examine the direction of expression changes after dasatinib treatment as compared to the baseline in GSE51083. Since the K-562 cell line had available expression measurements only at 2 hr but not at 6 or 24 hr after treatment with dasatinib in the NCI-TPW data, the direction of transcriptional changes of *HDAC* and *SIRT* genes in that cell line in the GSE51083 data was compared to the direction of consensus changes in the NCI-TPW dataset, as follows. After averaging the log_2_FC values among the multiple probes for each transcript and among the three replicate measurements in GSE51083, for those genes that satisfied the condition |log_2_FC| > 0.1, the direction of transcriptional change (positive or negative log_2_FC) was compared to the direction of consensus changes of log_2_FC in the NCI-TPW dataset for those genes which satisfied the criterion of concerted expression changes at 24 hr after treatment (Table 1).

**Table 1.** Concerted expression changes of *HDAC* and *SIRT* genes after drug treatment in the NCI-TPW dataset.

Gene	Aza	Bor	Cis	Das	Dox	Erl	Gel	Gem	Lap	Pac	Rap	Sor	Sun	Top	Vor
*HDAC1*	H6↓*, H24↓*, L24↓	H6↓, H24↓, L24↓*	L24↑		H24↓*		H6↓		H6↓			H24↓*, L24↓	H6↓	H6↑	H6↑, H24↑*, L6↑, L24↑*
*HDAC2*	H24↑, L24↑	L24↓		H6↓, H24↓, L24↓			H6↓, H24↓, L6↓		H24↓		H6↓	H24↓*	H24↓		
*HDAC3*	H6↓, H24↓	H6↓			H6↑, H24↓*	H24↓	H6↓		H24↓			L24↓	H24↓	H2↑, H6↑	H2↑*, H6↑*, H24↑*, L2↑, L6↑, L24↑
*HDAC4*	H2↓, H6↓*, L6↓*, L24↓*	H24↓***, L24↓**	H6↓*, H24↓**	H24↑*	H2↓*, H6↓**, H24↓***, L6↓*, L24↓	L2↓*,	H6↓*, H24↓**, L6↓*	H24↓*, L24↓*,						H2↓*, H6↓**, H24↓**	
*HDAC5*	H6↓*, H24↑*, L24↑	H24↑*	H24↑	H24↑*, L24↑*	H24↑**, L24↑	H24↑*		H24↑*, L24↑*	H6↑*, H24↑*, L24↑*		H6↑, H24↑*, L6↑, L24↑*	H6↑*, H24↑*, L6↑		H24↑*, L24↑	H6↑*, H24↑**, L6↑*, L24↑*
*HDAC6*	L24↑				H24↑			H24↓, L24↓		H24↑	H24↑, L24↑		H24↑,		H6↓, L6↓
*HDAC7*	H24↑, L24↑			H24↓*				H24↓							H6↓*, H24↓*, L6↓, L24↓
*HDAC9*	H6↑*, H24↑**	L24↑***		H6↓**, H24↓**, L24↓*	H2↓*, H6↓*, L24↑*	H24↑*		H24↑***, L2↓, L24↑***	H6↑*, H24↑*	L6↑		H6↑*, H24↑** L24↑*		H2↓*, H6↓**, H24↑**	H2↓**, H6↓*, L6↓*
*HDAC11*	L24↑			L24↑					L24↑*						
*SIRT1*	H6↑*, H24↑*, L6↑, L24↑*	H6↑*, H24↑*	H6↑, H24↑*		H2↑, H6↑*, H24↑*		H6↓, L6↓	H24↑*, L24↑*	H6↑*	H24↑	L2↓	L2↓		H2↓, H6↑*, H24↑*	H2↓*, L2↓, L6↓, L24↓*
*SIRT2*	H24↓*	H24↑*, L24↑*	H24↑*	L24↑*	H6↑, L24↑		H24↑*	H24↑, L24↑*	H24↑			H24↑*, L24↑*		H6↑*, H24↑*, L24↑	H6↑*, H24↑*, L6↑, L24↑*
*SIRT3*		H6↓, H24↓, L24↓		H24↑, L24↑	H24↑*						H24↑	L24↑	H24↑		H6↑, H24↑, L6↑
*SIRT4*	L24↓			L24↑	H6↑, H24↑*									H6↑*, H24↑*	H6↑*, H24↑*, L6↑*, L24↑*
*SIRT5*		H6↓, H24↓*, L6↓, L24↓		L24↑	H24↑	H24↑	H24↓				H24↑, L24↑	H24↑, L24↑	H24↑		H2↓, H6↓, L6↓, L24↓
*SIRT6*	H24↑*	H24↑*	H24↑		H24↑*		H24↓							H6↑*, H24↑*	
*SIRT7*	H24↑*, L24↑	H24↑*, L6↑*, L24↑*	H6↑*, H24↑		H24↑*		H6↓, L2↓, L6↓	H24↑*, L24↑*		H2↓	H6↓, H24↓, L2↓, L6↓, L24↓	H24↑*	H2↓, H6↑, H24↑	H6↑, H24↑*	H2↑*, H6↑*, H24↑*, L2↑*, L6↑*

Columns represent 15 agents in the NCI-TPW dataset. Aza, azacytidine; Bor, bortezomib; Cis, cisplatin; Das, dasatinib; Dox, doxorubicin; Erl, erlotinib; Gel, geldanamycin; Lap, lapatinib; Gem, gemcitabine; Pac, paclitaxel; Rap, rapamycin; Sor, sorafenib; Sun, sunitinib; Top, topotecan; Vor, vorinostat. Concerted changes in expression (↑, upregulated or ↓, downregulated) are shown for microarray experiments in which nearly all cell lines had a change in the same direction, with no more than 15 cell lines showing a change in the opposite direction. Expression changes are shown for the high (H) or low concentration (L) of each agent. The time when the change was observed is also indicated. Concerted changes for multiple conditions for the same drug-gene combination are separated by commas. High and low concentrations of each agent are listed in the Materials and Methods section.

* Concerted expression change as described above and log_2_FC (the difference of log_2_ expression values between treated and untreated cells) in that direction in some cell lines was ≥ 1 or ≤ −1.

** Concerted expression change and log_2_FC in that direction in some cell lines was ≥ 2.5 or ≤ −2.5.

*** Concerted expression change as described above and log_2_FC in that direction in some cell lines was ≥ 4 or ≤ −4.

For example, H↑24***, L↓2, L↑24*** for *SIRT9* after treatment with gemcitabine (Gem) indicates that it was upregulated in a concerted manner after treatment with both the high and low concentrations of that agent at 24 hours after treatment, with the change of log_2_ expression values in at least some cell lines being ≥ 4 or ≤ −4, and that it was also downregulated at 2 hours after treatment with the low concentration of gemcitabine, but the amplitude of the *SIRT9* expression changes under that condition did not reach ≥ 1 or ≤ −1.

GSE69395 [45] provided Affymetrix Human Genome U133 Plus 2.0 expression microarray data for the non-small cell lung cancer (NSCLC) cell lines A549, H661, H1666 and Cal12T, profiled at baseline and at 72 hr after treatment with 150 nM of dasatinib. The NCI-TPW condition closest to that data point was at 24 hr after treatment with the low concentration of dasatinib. Among the four cell lines in GSE69395, A549 is also a part of the NCI-60 cancer cell line panel.

GSE43010 [47] provided the data on changes in expression in transformed and non-transformed fibroblasts in response to vorinostat. It provided cell line data for BJ (normal fibroblasts) and BJ LTSTERas (transformed fibroblasts using the genes for the SV40 large T and small t antigens, hTERT and H-RAS). Neither cell line is a part of the NCI-60 panel. The data were provided at baseline and at 24 hr after treatment with 25 μM of vorinostat.

GSE84205 [46] included Affymetrix Human 1.0 STS expression microarray data for the 90-8TL malignant peripheral nerve sheath tumour (MPNST) cell line (not included the NCI-60 panel), profiled at baseline and at 24 hr after treatment with 2 μM of vorinostat. This is an intermediate concentration between the low and the high concentrations of vorinostat in the NCI-TPW dataset.

The direction of transcriptional changes of the *SIRT* genes was also validated using a published report by Kyrylenko et al. [7], who investigated the effects of multiple HDAC inhibitors on *SIRT* transcriptional changes in mouse neuroblastoma Neuro-2a cells, post-mitotic rat primary hippocampal and cerebellar granule neurons, and human SH-SY-5Y and Sk-N-As neuroblastoma cell lines at 12 and 24 hr after treatment. These authors used 1 µM of vorinostat in their experiments, which at 24 hr was comparable to the 24 hr time point of the low concentration of vorinostat in NCI-TPW. Other HDAC inhibitors used in their study included trichostatin A, n-butyrate, apicidin, and M344 [7].

The direction of HDAC and SIRT protein expression changes after treatment with vorinostat was examined using publicly available proteomic data. Such data were available for HL-60 acute myeloid leukaemia (AML) cells (also included in the NCI-60 panel) treated with 205 μM vorinostat vs DMSO, at 8 hr after treatment, from the study of Zhu et al. [42]. An additional dataset, PXD006005 from the PRIDE (Proteomics IDEntification Database) repository of the ProteomeXchange Consortium (https://www.ebi.ac.uk/pride/archive/projects/PXD006005), included the 72 hr time point for 10 μM vorinostat vs DMSO-treated fibroblasts carrying the NPC1^I1061T^ mutation representative of Niemann-Pick Type C1 disease, and was generated by Subramanian et al. [43].

Information about clinical trials which tested the dasatinib-vorinostat combination was obtained from the U.S. Library of Medicine ClinicalTrials.gov online database (https://clinicaltrials.gov) and biomedical publications.

### Cell lines

MDA-MB-231 (ATCC HTB-26) (RRID:CVCL_0062), IGROV-1 (RRID:CVCL_1304) and UACC-257 (RRID:CVCL_1779) were obtained from the NCI Developmental Therapeutics Program Tumor Repository. Each lot of cells was authenticated through a variety of molecular characterizations and identity was confirmed from frozen stock of each cell line using Identifiler DNA profiling [33,48]. Each cell line was tested for Mycoplasma using the MycoAlert Mycoplasma Detection Kit (Lonza).

Quality control information from the public datasets which were analysed in this study (Figure S1) was provided in the original reports, including the list of the NCI-60 cell lines profiled in the NCI-TPW dataset and detailed information about their authentication and Mycoplasma testing [33].

### Experimental validation of HDAC5 transcript and protein changes in the IGROV1, MDA-MB-231, and UACC-257 cell lines

We experimentally verified HDAC5 mRNA and protein expression changes in the IGROV1, UACC-257, and MDA-MB-231 cell lines after treatment with the high concentration (2 μM) of dasatinib. Total cell lysates were prepared from the cells which were untreated, vehicle control (DMSO) treated or treated with 2 μM of dasatinib. Transcriptional levels of *HDAC5* mRNA were measured at 24 hr after treatment using qRT-PCR using BioMark™ assay probes Hs00608351_m1 (the probe providing the best coverage, spanning exons 14 and 15) and Hs00608366_m1 (the most cited assay probe, spanning exons 3 and 4) (ThermoFisher Scientific/Life Technologies) and normalized to *GAPDH* expression which was stable across treatments. Protein levels were measured at 24 and 32 hr after dasatinib treatment. HDAC5 expression was measured using Western blot analysis, with 40 μg of lysate per lane run on a 3–8% Tris-Acetate gel for 65 minutes at 125 volts. Blots were probed with rabbit monoclonal antibody D1J7V (Cell Signaling TaqMan®) at 1:500. HDAC5 quantification was done using Licor Image Studio software and was first normalized to the levels of GAPDH loading controls and then divided by the levels from normalized untreated cells. Background was subtracted out based on an equal area (to bands) in the empty lane between the MWM and Untreated, next to GAPDH (the lane was loaded with loading buffer with lysis buffer). Duplicate biological replicate experiments were conducted on separate dates in order to confirm the consistency of mRNA and protein expression changes of HDAC5.

### Mutational status of the NCI-60 cell lines

Single nucleotide variant status of the NCI-60 cell lines for the *BRAF* gene was obtained from the NCI-60 whole exome sequencing project [49]. This information was downloaded from the CellMiner data download site (https://discover.nci.nih.gov/cellminer/loadDownload.do) [50].

### Analysis of regulatory microRNA expression changes after vorinostat treatment using data from the GEO dataset GSE69959

Multiple miRNAs have been implicated in the regulation of HDAC5 levels in malignant and non-malignant cells [51–58]. We investigated the changes in expression of such miRNAs after drug treatment. We examined the data in a publicly available GEO dataset, GSE69959 [59]. The miRNA expression data in that dataset had been generated with the help of the μParaflo microfluidic chip technology using a version of miRBase (Release 10.1). We examined these data to investigate expression changes of *miR-125a-5p, miR-589-5p, miR-9, miR-124*, and *miR-*217 in three AML cell lines, KASUMI1, U937, and K562, at 6 hr after treatment with 5 μM of vorinostat as compared to the pretreatment levels. The 6 hr time point and concentration are comparable to that time point and high concentration of vorinostat in NCI-TPW. Among the three cell lines, K-562 is a part of the NCI-60 panel. The data for an *miR-2861*, also implicated in HDAC5 regulation [51], were not available from that dataset. For each miRNA and each cell line, the log_2_-transformed normalized changes in expression of the treated vs untreated cell lines available from GEO were averaged among the 5 probes and 3 technical replicates. We verified miRNA annotation using the information from the RNAcentral database v.19 (https://rnacentral.org) [60] at the European Bioinformatics Institute and from the miRBase online database release 22.1 (https://www.mirbase.org) [61].

### Transcriptome-wide enrichment analysis of miRNA targeted genes

To examine the potential influence of miRNA regulation on drug response, gene set enrichment analyses of miRNA targets were performed using our in-house developed R package GSEA [62,63] (available for download at https://brb.nci.nih.gov/BRB-ArrayTools/ArrayToolsRPackages.html) and R v. 3.5.1. Our aim was to identify the miRNAs that were significantly enriched with targeted genes whose expression changes after treatment (relative to the untreated) in the NCI-TPW dataset were associated with response (log(GI50)) to dasatinib and vorinostat. We analysed NCI-TPW data for each of the following treatment conditions: dasatinib (high concentration at 24 hr after treatment) and vorinostat (high concentration, separately at 6 and 24 hr). Experimentally verified miRNA target lists were obtained from miRTarBase v. 7 [64,65], which contained data on 2599 human miRNAs. Association analyses between post-treatment gene expression changes and log(GI50) were performed using Spearman rank correlation. LS (log score) and KS (Kolmogorov-Smirnov) permutation tests were conducted to calculate a *p*-value measuring the gene set enrichment.

### Expression analysis of YAP1

Based on an earlier study that suggested a mediating effect of a master transcriptional regulator, YAP1 (YAP), on synergy between dasatinib and HDAC inhibitors [66], we investigated associations of its expression in the NCI-TPW dataset with cell line sensitivity to dasatinib and with transcriptional changes of *HDAC* and *SIRT* genes after dasatinib treatment. To assess the effect of dasatinib on *YAP1* expression, we evaluated concerted changes of *YAP1* using the same criteria as those for *HDAC* and *SIRT* genes. We used Spearman correlation to evaluate the association of log(GI50) of dasatinib with transcriptional changes of *YAP1* at 2, 6, and 24 hr after treatment with both concentrations of dasatinib. We also evaluated Spearman correlations of log(GI50) of dasatinib with median baseline expression of *YAP1* in untreated cell lines, computed across 15 agents at 6 hr. Finally, we evaluated a potential mediating role of YAP1 on changes in *HDAC* and *SIRT* expression induced by dasatinib. We analysed Spearman correlation of log_2_FC of *HDAC* and *SIRT* genes at 6 and 24 hr after treatment with the high concentration of dasatinib with baseline expression of *YAP1* and with log_2_FC of *YAP1* at those time points.

### Expression analysis of dasatinib kinase target genes

Since dasatinib is a broad multi-kinase inhibitor [67], we used the NCI-TPW dataset to investigate potential influences of its kinase targets on *HDAC* and *SIRT* expression. To achieve this goal, we examined the effects of dasatinib treatment on mRNA expression of 22 genes (*ABL1, ABL2, ZAK, BCR, BTK, CSF1R, CSK, EPHA2, EPHA5, EPHB4, FGR, FRK, FYN, KIT, LCK, LYN, PDGFRA, PDGFRB, MAP4K5, MAPK14, SRC*, and *YES1*) encoding dasatinib targets with kinase activity. Information about dasatinib targets was obtained from the DrugBank Online portal (https://go.drugbank.com) [68] and biomedical publications [31,67,69–73]. We also analysed Spearman correlations of baseline expression of dasatinib kinase target genes and of their post-treatment expression changes (log_2_FC) with log(GI50) of dasatinib and with log_2_FC of *HDAC* and *SIRT* genes at 6 and 24 hr after treatment with the high concentration of dasatinib. The *p*-values from Spearman correlation analyses of dasatinib kinase target genes were adjusted for multiple testing using the FDR adjustment, which was applied to a combined set of *p-*values from the analyses of different time points and from baseline correlation analysis.

## Results

### Drug treatment induced multiple concerted changes in HDAC and SIRT gene expression

Table 1 provides the list of all conditions under which each *HDAC* and each *SIRT* gene satisfied the criteria of concerted transcriptional changes after drug treatment in the NCI-TPW dataset. As noted previously [33,34], higher drug concentrations and later time points (6 hr and especially 24 hr after treatment) more often resulted in concerted transcriptional changes. Each of the 15 antitumor agents induced concerted changes in multiple genes. Notably, *HDAC4* and *HDAC9* not only showed concerted changes in response to multiple agents, but they also had high amplitudes of transcriptional response to several agents (|log_2_FC| ≥ 2.5 or 4 in some cell lines; Table 1; Figure S2). *HDAC4* was downregulated in all cases of concerted changes, whereas the expression of *HDAC9* was either decreased or increased in a concerted manner for different agents, time points, and concentrations (Table 1).

The non-selective HDAC inhibitor vorinostat and the DNMT1 inhibitor azacytidine induced concerted transcriptional effects in multiple *HDAC* and *SIRT* genes, consistent with broad epigenetic effects of both agents [25,36,74–77]. Treatment with vorinostat led to pronounced changes in expression of multiple *HDAC* and *SIRT* genes (Table 1). *HDAC6*, *HDAC7*, *HDAC9*, *SIRT1*, and *SIRT5* were downregulated in a concerted manner after treatment with vorinostat under multiple conditions. Interestingly, expression of *HDAC1, HDAC3, HDAC5, SIRT2, SIRT3, SIRT4*, and *SIRT7* was upregulated in a concerted manner after treatment with vorinostat (Table 1; Figure S3). As the products of *HDAC1* and *HDAC3* are enzymatic targets of vorinostat [2], the effect of vorinostat on the increase in their mRNA levels was in the opposite direction from its inhibition of the enzymatic activity of both HDACs.

**Figure 1.**
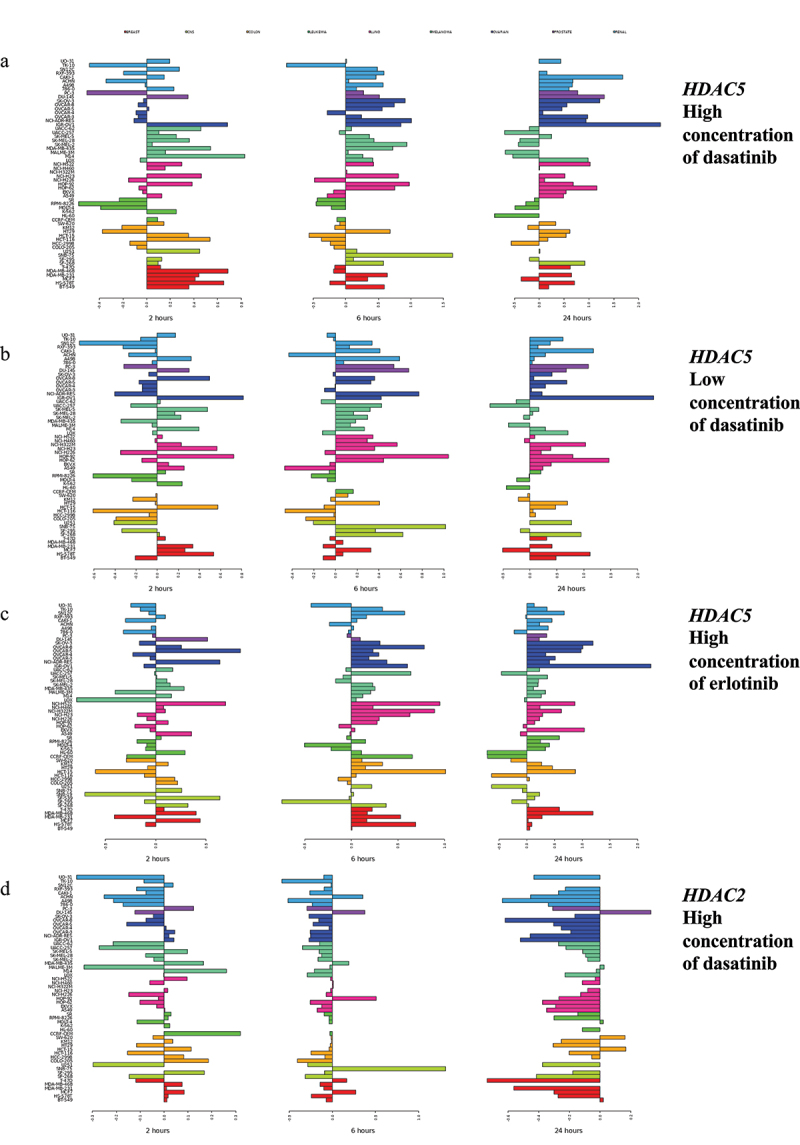
Examples of changes in expression of *HDAC* genes in response to treatment with erlotinib and dasatinib. Shown are transcriptional changes (log_2_FC) at 2 (left panel), 6 (middle panel), and 24 hr (right panel) after treatment. Horizontal right bars indicate elevated gene expression, whereas left bars show decreased expression relative to the untreated cell lines. As summarized in Table 1, concerted upregulation at 24 hr (shown on the right most panels) was observed for *HDAC5* after treatment with (a) the high (2,000 nM) and (b) the low (100 nM) concentrations of dasatinib and (c) the high (10,000 nM) concentration of erlotinib. (d) expression changes of *HDAC2* after treatment with the high concentration of dasatinib showed concerted downregulation at 6 hr (middle panel) and 24 hr (right panel). Colors represent cancer categories. The scale on the bottom represents log_2_ difference between expression values of treated and untreated cell lines. The scale for each microarray experiment is specific to that experiment.

Treatment with non-epigenetic agents with various mechanisms of action also induced concerted expression changes in multiple *HDAC* and *SIRT* genes (Table 1; Figure 1; Figure S4). For example, at 24 hr after treatment with the high or low concentration of the multi-kinase inhibitor dasatinib, *HDAC4, HDAC5, HDAC11, SIRT2, SIRT3, SIRT4*, and *SIRT5* were upregulated in a concerted manner, while *HDAC2, HDAC7*, and *HDAC9* were downregulated. *HDAC2* and *HDAC9* were also downregulated at 6 hr after treatment with the high concentration of dasatinib.

### Correlation of increased mRNA expression of HDAC5, HDAC1, and SIRT genes after drug treatment with higher sensitivity to kinase inhibitors

Table 2 provides the list of significant associations satisfying *p*_FDR_ <0.05 between changes in expression (log_2_FC) of *HDAC* and *SIRT* genes and cell line sensitivity (log(GI50)) to the antitumor agents profiled in NCI-TPW. An expanded list of associations satisfying the less stringent threshold, *p*_FDR_ <0.1, is provided in Supplementary Table S1. Unexpectedly, we observed strong significant associations of *HDAC5* upregulation at 24 hr with increased sensitivity to the ABL/SRC family multi-kinase inhibitor dasatinib and the EGFR inhibitor erlotinib (negative correlation between log_2_FC and log(GI50), with −0.7806 ≤ Spearman correlation coefficient ρ  ≤ −0.4003, 3.74 × 10^−11^ ≤ *p*_0_ ≤0.0017, 6.28 × 10^−8^ ≤ *p*_FDR_ ≤0.0877; Figure S5, Table 2, and Supplementary Table S1). The strongest associations were between sensitivity to dasatinib and the amplitude of transcriptional upregulation of *HDAC5* at 24 hr after treatment (ρ  = −0.7806 and −0.6871, *p*_FDR_ = 6.28 × 10^−8^ and 2.17 × 10^−5^ for the high and low concentrations, respectively; Table 2). *HDAC5* upregulation also showed a trend for an association with increased sensitivity to the EGFR/HER2 inhibitor lapatinib, however it did not reach significance after FDR adjustment for multiple testing (ρ  = −0.3648 and −0.2920, *p*_0_ = 0.0067 and 0.0305, *p*_FDR_ ≥0.1564 for the high and low concentrations, respectively; data not shown). *HDAC5* was upregulated in a concerted manner at 24 hr after treatment with the high and low concentrations of dasatinib and lapatinib and with the high concentration of erlotinib (Table 1; Figure 1).

**Table 2.** Spearman correlations between log(GI50) and log_2_FC of HDAC and SIRT genes satisfying *p*_FDR_ <0.05.

Agent	Gene	Condition	Spearman ρ	*p*_0_	*p*_FDR_
Dasatinib	*HDAC5*	High 24	−0.7806	3.74 × 10^−11^	6.28 × 10^−8^
Dasatinib	*HDAC5*	Low 24	−0.6871	2.58 × 10^−8^	2.17 × 10^−5^
Dasatinib	*HDAC2*	High 24	0.5774	1.40 × 10^−5^	0.00336
Dasatinib	*SIRT1*	High 6	−0.5031	9.05 × 10^−7^	0.01382
Dasatinib	*SIRT2*	High 24	−0.5158	0.00015	0.01925
Dasatinib	*HDAC1*	Low 6	−0.4628	0.00037	0.04187
Dasatinib	*HDAC1*	High 6	−0.4551	0.00048	0.04490
Erlotinib	*HDAC5*	High 24	−0.4625	0.00023	0.02714
Erlotinib	*SIRT5*	High 24	−0.4429	0.00044	0.04377
Cisplatin	*HDAC4*	High 24	0.6251	3.37 × 10^−7^	0.00019
Cisplatin	*SIRT1*	High 24	−0.5730	4.83 × 10^−6^	0.00190
Cisplatin	*HDAC1*	High 24	0.4913	0.00014	0.01925
Gemcitabine	*HDAC1*	Low 24	0.4595	0.00042	0.04377
Topotecan	*HDAC1*	High 24	0.5429	1.07 × 10^−5^	0.00300
Geldanamycin	*HDAC4*	Low 24	0.5653	5.65 × 10^−6^	0.00190
Azacytidine	*HDAC9*	High 24	−0.6136	3.27 × 10^−5^	0.00687
Paclitaxel	*HDAC9*	Low 24	0.4920	6.53 × 10^−5^	0.01219

The results are grouped by agent and sorted by *p*_FDR_.

ρ, Spearman correlation coefficient.

Condition indicates concentration (high or low) and the time point (6 or 24 hr after treatment). None of the changes at 2 hr after treatment satisfied *p*_FDR_ < 0.05.

*p*_0_, *p*-value prior to the FDR adjustment.

*p*_FDR_, FDR adjusted *p*-value.

*HDAC1* expression did not satisfy the criterion for concerted changes after dasatinib treatment (Table 1; Figure S4A). However, similar to *HDAC5*, log_2_FC of *HDAC1* was negatively correlated with log(GI50) of dasatinib, indicating that cell lines with increased *HDAC1* expression after treatment were more sensitive, and cell lines with decreased *HDAC1* expression were more resistant to dasatinib (at 6 hr after treatment with the high or the low concentration, and at 24 hr at the low concentration; −0.4650 ≤ ρ  ≤ −0.4551, 0.0419 ≤ *p*_FDR_ ≤0.0518; Table 2, Supplementary Table S1). In contrast to *HDAC5*, changes in *HDAC1* expression were not associated with sensitivity to either erlotinib or lapatinib (data not shown). The association of upregulation of *HDAC5* with sensitivity to multiple kinases and of *HDAC1* with dasatinib sensitivity is unexpected, based on frequently reported clinical benefits of inhibition of their products in cancer treatment.

Upregulation of several *SIRT* genes was also significantly associated with sensitivity to dasatinib, with a negative correlation between log_2_FC and log(GI50). An increase in *SIRT1* expression at 6 hr after treatment with both the high and the low concentrations of dasatinib and upregulation of *SIRT2, SIRT3, SIRT4*, and *SIRT5* at 24 hr after treatment with the high concentration were associated with dasatinib sensitivity (−0.5158 ≤ ρ  ≤ −0.4234, 9.05 × 10^−5^ ≤ *p*_FDR_ ≤0.0909; Table 2; Supplementary Table S1). Among them, only *SIRT3* satisfied the criterion for concerted upregulation after treatment with the high concentration of dasatinib at 24 hr (Figure S4C). *SIRT2, SIRT3, SIRT4*, and *SIRT5* had concerted upregulation by the low concentration of dasatinib at 24 hr (Table 1; Figure S4). In addition to the associations of *SIRT* genes with sensitivity to dasatinib, *SIRT5* had concerted upregulation at 24 hr after treatment with the high concentration of erlotinib, which also associated with erlotinib sensitivity (ρ  = −0.4429; *p*_FDR_ = 0.0438; Tables 1 and 2; Figure S4D). Associations of upregulation of *SIRT3* and *SIRT4* with dasatinib sensitivity were consistent with previously reported protective roles of both genes in the treatment of hepatocellular carcinoma and with the positive effect of *SIRT3* upregulation on HCC sensitivity to a variety of antitumor agents, including the kinase inhibitors sorafenib and regorafenib [32]. By contrast, associations of stronger upregulation of *SIRT1, SIRT2*, and *SIRT5* with sensitivity to dasatinib were unexpected, since previous studies had reported benefits of their downregulation or depletion in improving cancer outcomes [13,14,78,79]. Notably, an improvement in dasatinib sensitivity had been reported in melanoma cell lines with *SIRT2* knockdown [31].

In contrast to the associations of post-treatment upregulation of *HDAC1, HDAC5, SIRT1, SIRT2, SIRT3, SIRT4*, and *SIRT5* with dasatinib sensitivity, *HDAC2* was downregulated by dasatinib in a concerted manner at 6 hr (high concentration) and 24 hr (both concentrations; Table 1), and the amplitude of *HDAC2* downregulation at 24 hr after treatment with the high concentration was significantly associated with sensitivity to dasatinib (positive correlation between log_2_FC and log(GI50) with ρ  = 0.5774 and *p*_FDR_ = 0.0034; Table 2, Supplementary Table S1).

While the majority of the *HDAC* and *SIRT* expression changes were associated with log(GI50) of the kinase inhibitors dasatinib and erlotinib, we also observed significant associations between changes in mRNA expression and cell line response to other antitumor agents with different mechanisms of action (Table 2; Supplementary Table S1). Some associations were similar to the patterns observed for *HDAC5* and *HDAC1* with kinase inhibitors, suggesting a surprising association of post-treatment transcriptional upregulation with higher sensitivity to specific agents. Other trends suggested benefits of downregulation of specific genes for improved drug sensitivity. While, as described above, increased mRNA expression of *HDAC1* after treatment was associated with sensitivity to dasatinib, *HDAC1* associations with response to DNA damaging agents were in the opposite direction. The correlations between log_2_FC of *HDAC1* at 24 hr and log(GI50) of cisplatin, gemcitabine, and topotecan were significantly positive (0.4595 ≤ ρ  ≤0.5429, *p*_FDR_ ≤0.0438; Table 2), indicating that cell lines with a greater increase in expression after treatment were more resistant to these DNA damaging agents, while the cell lines where *HDAC1* expression was more strongly downregulated were more sensitive to them. This direction of the correlation with DNA damaging agents is consistent with previously reported benefit of diminished *HDAC1* expression in neuroblastoma cell lines, which increased their sensitivity to etoposide [29]. Similarly, log_2_FC of *HDAC4* was positively associated with log(GI50) of cisplatin at 6 hr (both concentrations) and 24 hr (high concentration) and with response to geldanamycin at 24 hr after treatment with the low concentration (0.4095 ≤ ρ  ≤0.6251, 0.0002 ≤ *p*_FDR_ ≤0.0877; Table 2; Supplementary Table S1). *HDAC4* was downregulated by both cisplatin and geldanamycin in a concerted manner (Table 1), and cell lines with its stronger downregulation were more sensitive to these agents. At 24 hr, log_2_FC of *HDAC2* was positively correlated with log(GI50) of doxorubicin (low concentration, ρ  = 0.4272, *p*_FDR_ = 0.0597), and both *HDAC2* and *HDAC9* had positive correlations with log(GI50) of paclitaxel (low concentration, ρ  = 0.4272 and 0.4290, *p*_FDR_ = 0.0558 and 0.0122, respectively; Table 2; Supplementary Table S1), suggesting that cell lines where such genes were downregulated after treatment were more sensitive to these agents, and cell lines with upregulated genes were more resistant. In contrast, *HDAC9* was downregulated by azacytidine at 6 and 24 hr in a concerted manner and showed a significant negative correlation between log_2_FC and log(GI50) at 24 hr (high concentration, ρ = −0.6136, *p*_FDR_ = 0.0069; Tables 1 and 2). This indicated that the cell lines in which *HDAC9* was more strongly downregulated by azacytidine had a tendency to be more resistant to that agent. The amplitude of expression changes of *SIRT2, SIRT3*, and *SIRT7* showed a similarly negative association with log(GI50) of cisplatin and geldanamycin at 24 hr (high concentrations, −0.5730 ≤ ρ  ≤ −0.4191, 0.0019 ≤ *p*_FDR_ ≤0.0810; Table 2 and Supplementary Table S1).

### Associations of baseline expression levels with drug response

Associations between baseline expression and drug response satisfying *p*_FDR_ <0.1 are provided in Supplementary Table S2. Elevated baseline expression of *HDAC1* was associated with higher sensitivity to sunitinib and satisfied *p*_FDR_ <0.05 (ρ  = −0.4463, *p*_FDR_ = 0.0468), whereas increased baseline expression of *HDAC7* was associated with sensitivity to dasatinib (ρ  = −0.4117, *p*_FDR_ = 0.0769). This direction of correlations of increased expression of *HDAC1* and *HDAC7* in untreated cell lines with sensitivity to kinase inhibitors was similar to the direction of associations observed after drug treatment for upregulation of *HDAC5* with dasatinib and erlotinib sensitivity and of *HDAC1* with dasatinib sensitivity (Table 2 and Supplementary Table S1). In contrast, associations of baseline expression of *HDAC7* with response to paclitaxel and of *SIRT6* with response to erlotinib were positive (ρ  = −0.4188 and −0.4111, *p*_FDR_ = 0.0583 and 0.0662, respectively).

### Validation of HDAC and SIRT mRNA and protein expression changes after treatment with dasatinib and vorinostat in public datasets

To confirm mRNA and protein expression changes of HDACs and SIRTs genes in response to dasatinib and vorinostat, we examined publicly available datasets from NCBI GEO and biomedical literature. Even though only a subset of the cell lines in independent datasets were also a part of the NCI-60 panel, and although some drug concentrations or post-treatment time points in separate datasets were not identical to those in NCI-TPW, both mRNA and protein expression changes after treatment were predominantly in agreement with directions of concerted transcriptional changes in NCI-TPW data.

Supplementary Table S3A and Supplementary Table S3B show transcriptional changes in *HDAC* and *SIRT* genes and isoforms in response to dasatinib in two public datasets, as compared to the concerted changes in NCI-TPW. These tables show the directions of transcriptional changes in the K-562 CML cell line in response to dasatinib at 24 hr after treatment in the NCBI GEO dataset GSE51083 [44] (Supplementary Table S3A) and in four NSCLC cell lines in GSE69395 [45] at 72 hr after treatment with 150 nM of dasatinib (Supplementary Table S3B). These directions were compared to the direction of concerted changes in the NCI-TPW consensus. Both tables show that when considering the changes after treatment above the minimal threshold of |log_2_FC| > 0.1, transcription of a number of *HDAC* and *SIRT* genes in individual cell lines from independent datasets was upregulated or downregulated in the same direction with concerted changes of the NCI-TPW consensus. In Supplementary Table S3A, the direction of transcriptional changes was the same in GSE51083 and NCI-TPW for each *HDAC* and *SIRT* gene satisfying both criteria (|log_2_FC| > 0.1 in GSE51083 and concerted changes in NCI-TPW). However, there was a considerable variation among cell lines in the magnitude and direction of expression changes for individual genes. Of note, in the GSE69395 dataset *HDAC5* was most strongly upregulated (log_2_FC = 0.2789) in the Cal12T cell line. This cell line is not a part of the NCI-60 panel but has been reported to be sensitive to dasatinib [45,71]. However, GSE69395 provided data at 72 hr after treatment, as opposed to the latest time point in NCI-TPW being 24 hr, resulting in a partial agreement with the direction of changes in NCI-TPW. Due to time dependency of transcription and mRNA degradation, these datasets may have underlying biological differences between mRNA levels at different time points after treatment.

Transcriptional changes in response to vorinostat are summarized in Supplementary Table S4. Supplementary Table S4A compares log_2_FC of transcriptional changes in *HDACs* and *SIRTs* in response to vorinostat in GSE43010 [47] to concerted changes in NCI-TPW at 24 hr in both datasets. The data in GSE43010 had been generated after treatment with 25 μM of vorinostat, which is substantially higher than the high concentration (5 μM) of vorinostat in NCI-TPW. GSE43010 included measures for the normal fibroblast cell line, BJ, and the transformed fibroblast cell line, BJ LTSTERas, neither of which were a part of the NCI-60 panel. The changes in expression of many *HDAC* and *SIRT* genes in GSE43010 were consistent with those observed in the NCI-60 cancer cell lines in NCI-TPW. Of note, while vorinostat induced the same directions of transcriptional changes in both transformed and non-transformed fibroblasts for the majority of *HDAC* and *SIRT* genes, the amplitude of their transcriptional upregulation or downregulation was higher in the transformed fibroblast cell lines BJ LTSTERas.

Supplementary Table S4B compares the changes in the MPNST cell line 90-8TL from GSE84205 [46], at 24 hr after treatment with 2 μM of vorinostat, to the direction of changes in the NCI-TPW consensus at 24 hr after treatment with the high or low concentrations of vorinostat. Even though fibroblast or MPNST cell lines were not a part of the NCI-60 panel, there was an agreement between the directions of expression changes of many *HDAC*s and some *SIRT*s in response to vorinostat in GSE84205 and in NCI-TPW. *HDAC1, HDAC3, HDAC5*, and *SIRT2* were consistently upregulated, and *HDAC7, HDAC9*, and *SIRT1* were consistently downregulated by vorinostat in all three datasets (GSE43010, GSE84205, and NCI-TPW consensus).

Concerted transcriptional changes of *SIRT* genes in NCI-TPW were also in agreement with the results of Kyrylenko et al. [7], who examined the effects of HDAC inhibitors on mouse and human neuroblastoma cell lines and on non-malignant post-mitotic rat primary hippocampal and cerebellar granule neurons. Concerted transcriptional upregulation of *SIRT2* and *SIRT4* and concerted downregulation of *SIRT1* and *SIRT5* by vorinostat in NCI-TPW were in agreement with the direction of their changes induced by trichostatin A and n-butyrate, reported by Kyrylenko et al. [7]. These authors further confirmed upregulation of *SIRT2* at 12 and 24 hr in mouse neuroblastoma cells Neuro-2a in response to the HDAC inhibitors vorinostat, apicidin, and M344.

The direction of previously reported changes for HDAC and SIRT protein levels in malignant and non-malignant cells after treatment with vorinostat was in most cases in agreement with the direction of concerted transcriptional changes in NCI-TPW. Zhu et al. [42] used SILAC protein quantification labelling, basic HPLC fractionation, and LC-MS/MS analysis to provide global proteome measurements for the AML cell line HL-60, at 8 hr after treatment with 205 μM vorinostat, as compared to DMSO. While HL-60 is also a part of the NCI-60 panel profiled in NCI-TPW, however the two studies did not use identical conditions, as the 8 hr time point was not included in the NCI-TPW measures, and the 205 μM concentration of vorinostat vastly exceeded the high concentration of vorinostat of 5 μM in NCI-TPW. Three HDAC and SIRT proteins had available relative abundance ratios (M/L) of vorinostat-treated to DMSO-treated cells generated by Zhu et al. [42]. These were HDAC1, HDAC2, and SIRT5 (M/L ratio = 1.1608, 1.5431, and 1.0042, and posterior error probability PEP = 1.3402 × 10^−112^, 7.0097 × 10^−136^, and 4.3668 × 10^−10^ for each of the three proteins, respectively). Whereas HDAC2 did not satisfy the criterion for concerted expression changes in NCI-TPW, and the protein abundance of SIRT5 was essentially unchanged between treated and untreated cells in the study of Zhu et al. [42], upregulation of HDAC1 protein levels by vorinostat was in agreement with its concerted transcriptional upregulation in NCI-TPW at 6 and 24 hr using the high and low concentrations of vorinostat. Upregulation of HDAC1, an enzymatic target of vorinostat inhibition, at both mRNA and protein levels is notable.

An additional set of measures of changes in protein abundance in non-malignant cells was generated by a global MS/MS proteomic study of Subramanian et al. [43]. Their measures were obtained at the 72 hr time point for 10 μM vorinostat vs DMSO treated fibroblasts with the NPC1^I1061T^ mutation representing a model for a progressive neurodegenerative disorder, Niemann-Pick Type C1 disease. Among other proteins that study generated relative proteomic abundance data (represented as z-scores) for HDAC1, HDAC2, HDAC3, HDAC4, HDAC7, and SIRT2, comparing vorinostat-treated vs DMSO-treated cells. Each of these proteins had measures for 1 or 2 out of three replicate experiments. *HDAC2* and *HDAC4* did not satisfy the criteria for concerted expression changes in NCI-TPW after treatment with vorinostat and were not compared to the proteomic data. Changes in protein abundance of HDAC1 were not in agreement with NCI-TPW, as it was slightly downregulated in the proteomic study of NPC1 fibroblasts (z-scores of −0.30 and −0.02 in two replicate experiments), as opposed to the concerted upregulation of *HDAC1* mRNA levels in NCI-TPW. In contrast, the changes in abundance of HDAC3, HDAC7 and SIRT2 after vorinostat treatment were in agreement with transcriptional changes in NCI-TPW (z-score = 0.68 for HDAC3, showing its protein upregulation despite being an enzymatic target for vorinostat, −1.50 for HDAC7 indicating its downregulation, and 0.29 and 0.12 in two replicate experiments for SIRT2, showing its upregulation). Despite differences between the time points and vorinostat concentrations in the two studies, transcriptional and proteomic changes in expression of HDAC3, HDAC7, and SIRT2 were consistent and appear to be induced in both malignant and non-malignant cells. As discussed above, upregulation of SIRT2 protein levels by vorinostat was also consistent with the increase of *SIRT2* mRNA expression in three separate independent studies [7,46,47].

### Experimental validation of HDAC5 expression changes after dasatinib treatment

To experimentally validate mRNA and protein expression changes of HDAC5 after treatment with vorinostat, we selected three cell lines with different levels of sensitivity to dasatinib and different amplitudes and directions of transcriptional changes at 24 hr after treatment with the high concentration of dasatinib in NCI-TPW. All three cell lines had some baseline expression of *HDAC5* in untreated cell lines. IGROV1 is an ovarian carcinoma cell line which was highly sensitive to dasatinib (log(GI50) = −8 in NCI-TPW, representing the highest possible sensitivity based on the screening range), and it showed a strong upregulation of *HDAC5* by dasatinib (log_2_FC = 2.437 in NCI-TPW, baseline expression in untreated cells = 4.551 measured at 6 hr and 4.335 at 24 hr). MDA-MB-231 is a triple negative breast cancer cell line which was also sensitive to dasatinib (log(GI50) = −7.728 in NCI-TPW). Sensitivity of MDA-MB-231 to dasatinib was also previously established by earlier studies [80–83]. It had low to moderate *HDAC5* upregulation after treatment (log_2_FC = 0.648 in NCI-TPW, with baseline expression = 4.419 at 6 hr and 4.872 at 24 hr). The MDA-MB-231 cell line has been widely used as a model in studies of the role of HDAC5 in cancer and of its effect on tumour responses to a variety of antitumor agents [22,23,54,84–88]. As a negative control, we used UACC-257, a melanoma cell line which was resistant to dasatinib (log(GI50) = −4.952) and was low to moderately downregulated by the high concentration of dasatinib at 24 hr (log_2_FC = −0.696, baseline expression = 4.701 at 6 hr and 4.978 at 24 hr).

**Figure 2.**
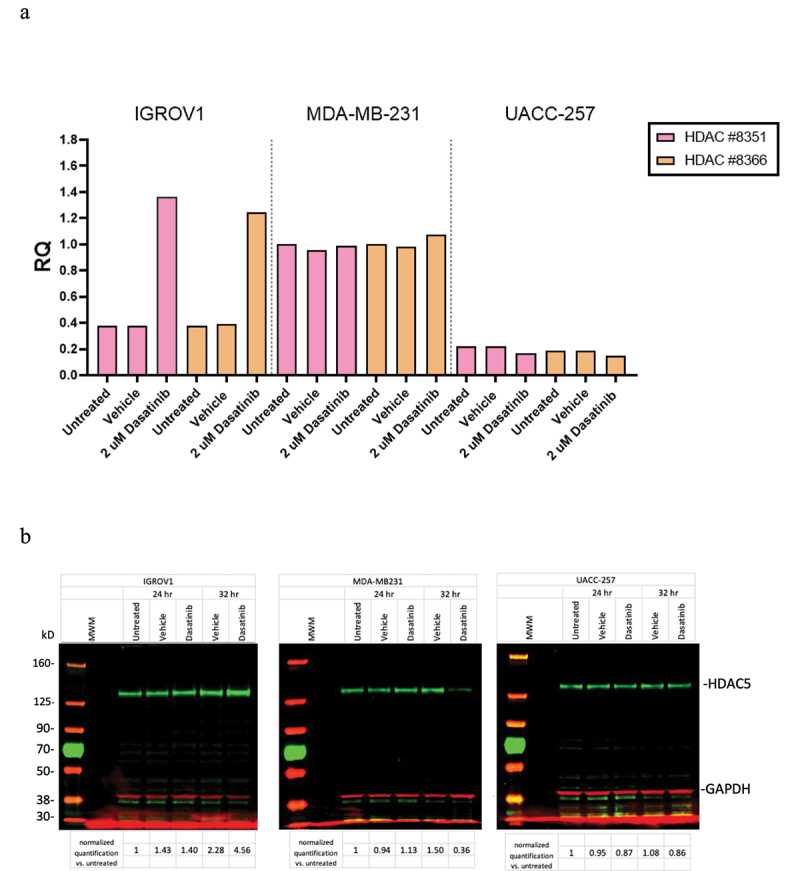
Experimental validation of transcriptional and protein levels of HDAC5 in the IGROV1, MDA-MB-231, and UACC-257 cell lines after treatment with 2000 nM of dasatinib. (a) Transcriptional changes in mRNA levels using RT-PCR. Results of two independent probes to *HDAC5* are normalized to GAPDH. (b) Changes in protein levels using Western blots. Protein results are first normalized to GAPDH and then values divided by normalized untreated cells. Full blots are shown.

Our duplicate experiments measuring mRNA and protein levels of HDAC5 in these three cell lines were in general agreement with the observed direction and magnitude of transcriptional changes in the NCI-TPW dataset. They consistently confirmed upregulation of HDAC5 mRNA levels at 24 hr and protein expression at 24–32 hr after treatment with 2000 nM of dasatinib in the IGROV1 cell line. The fold changes (RQ) in mRNA levels and protein levels of HDAC5 normalized to GAPDH from one of the experiments are shown in Figures 2(a,b). In that experiment, at 24 hr mRNA levels of *HDAC5* in the IGROV1 cell line after treatment with dasatinib were considerably increased (Figure 2(a)), and at 32 hr the level of HDAC5 protein in that cell line was also considerably increased (Figure 2(b)). In contrast to IGROV1, in the MDA-MB-231 cell line the mRNA levels of *HDAC5* remained relatively stable after dasatinib treatment, and the protein levels were slightly upregulated at 24 hr but not at 32 hr after treatment, whereas both mRNA and protein levels of HDAC5 in UACC-257 were slightly downregulated. Although we did not observe a consistent low to moderate upregulation of MDA-MB-231 on the mRNA levels which occurred in the NCI-TPW experiments, all other mRNA and protein changes in our experimental analysis were consistent with the direction and the strength of response in HDAC5 regulation in sensitive and resistant cell lines noted in the NCI-TPW data.

### Vorinostat-induced changes in expression of miRNAs with potential regulatory effects on HDAC5

In a variety of normal and cancer cells, HDAC5 levels are regulated by multiple miRNAs including *miR-125a-5p, miR-589-5p, miR-2861, miR-9, miR-124*, and *miR-*217 [51–58]. We used the publicly available dataset GSE69959 [59] to investigate expression changes of those miRNAs in the AML cell lines KASUMI1, U937, and K562, at 6 hr after treatment with 5 μM of vorinostat as compared to the pretreatment levels. The 6 hr time point and concentration correspond to the high concentration of vorinostat in NCI-TPW, and among the three cell lines K-562 is a part of the NCI-60 panel.

The changes in expression of the five miRNAs with available data in GSE69959, averaged among the probes and technical replicates, are summarized in Figure S6. *miR-125a-5p, miR-589-5p*, and *miR-*217 were upregulated by vorinostat in all three cell lines. In contrast, *miR-9* and *miR-124* had a mixed response to vorinostat. They were upregulated only in a subset of the cell lines and were downregulated or had no expression change in other cell lines. No measurements were available for *miR-2861.*

### miRNA regulons enriched with targeted genes associated with drug response

Transcriptome-wide enrichment analysis using NCI-TPW data showed that the genes regulated by *miR-125a-5p, miR-589-5p*, and *miR-*217 were significantly or nearly significantly associated with response to the high concentration of dasatinib at 24 hr whether using the LS or the KS statistics (0.0005 ≤ *p* ≤ 0.0567; Supplementary Table S5). The genes targeted by *miR-2861* were nearly significantly associated with dasatinib response in the analysis that used the KS statistic (*p* = 0.0529), however they did not show a significant association when using the LS statistic (*p* = 0.1458). The gene sets targeted by *miR-124-5p, miR-124-3p, miR-9-5p* and *miR-9-3p* were not significantly associated with response to the high concentration of dasatinib at 24 hr (*p* ≥ 0.13 in all analyses).

No association with cell line response to the high concentration vorinostat at 6 or 24 hr was observed for the target genes of any of the six miRNAs. The lack of association with vorinostat response and the association with dasatinib response may be consistent with the potential roles of *miR-125a-5p, miR-589-5p*, and *miR-*217 in HDAC5 regulation. Our gene set enrichment analysis of miRNA targeted genes identified those miRNAs, for which their target genes were associated with response to each agent. Upregulation of *HDAC5* after treatment was significantly associated with cell line sensitivity to dasatinib (Tables 1 and 2; Figure 1), consistent with the association of the target genes for these three miRNAs. Even though *HDAC5* was upregulated by vorinostat in a concerted manner, as were *125a-5p, miR-589-5p*, and *miR-*217, the increase in *HDAC5* expression was not associated with response to vorinostat, consistent with the lack of association of the target genes for these miRNAs (Table 1; Supplementary Table S1; Figure S3).

### YAP1 expression and transcriptional response to dasatinib

Yang et al. suggested a potential role of the master regulator YAP1 in sensitivity of tumour cells to dasatinib and in synergy between HDAC inhibitors and dasatinib [66]. They found high mRNA and protein expression of YAP1 to be associated with increased dasatinib sensitivity and with increased resistance to HDAC inhibitors including vorinostat. Consistent with their findings, baseline expression of *YAP1* in the NCI-TPW dataset had a very weak trend for association with sensitivity to dasatinib and with resistance to vorinostat (Spearman ρ = −0.1927 and 0.1530, respectively; not statistically significant with *p*_0_ ≥0.1305). *YAP1* showed concerted downregulation at 6 and 24 hr by the high concentration of dasatinib, and by both concentrations of vorinostat (Figure S7). A lesser degree of *YAP1* downregulation by dasatinib was weakly associated with dasatinib sensitivity at 6 hr or 24 hr (ρ = −0.2627, −0.2326, and −0.1302 at 6 hr/low, 24 hr/low, and 24 hr/high concentrations, *p*_0_ = 0.0527, 0.1005, and 0.3728, respectively; not significant with *p*_*FDR*_ ≥0.3518). While these trends supported the previously reported [66] association of *YAP1* expression with dasatinib sensitivity, *YAP1* expression was not significantly associated with expression changes of any *HDAC* or *SIRT* genes at 6 or 24 hr after treatment with the high concentration of dasatinib (*p*_*FDR*_ ≥0.1554; Supplementary Table S6). Although they were non-significant after adjustment for multiple testing, changes in *YAP1* expression after dasatinib treatment were weakly negatively correlated with log_2_FC of *HDAC2* and weakly positively correlated with log_2_FC of *SIRT1, SIRT3*, and *SIRT4* (Supplementary Table S6). These correlations were in agreement with the directions of associations of log_2_FC of *HDAC2, SIRT1, SIRT3*, and *SIRT4* with dasatinib sensitivity (Supplementary Table S1). However, correlations of these genes with downregulation of *YAP1* were weak (0.2251 ≤ |ρ | ≤ 0.3059; 0.0326 ≤ *p*_0_ ≤0.0952), whereas their correlation with log(GI50) of dasatinib were strong and statistically significant. Neither baseline expression nor log_2_FC of *YAP1* was correlated with log_2_FC of additional genes (*HDAC5, HDAC1, SIRT2*, and *SIRT5*; Supplementary Table S1) which were associated with dasatinib response (data not shown). These results suggest that if YAP1 is involved in dasatinib response, it may not be the main driver of the strong associations of expression changes of many *HDAC* and *SIRT* genes with dasatinib sensitivity. Upregulation of *HDAC1, HDAC5, SIRT2*, and *SIRT5* and its potential effect on dasatinib sensitivity are likely to be influenced by other factors, which remain to be determined.

### Expression of dasatinib kinase target genes was associated with transcriptional changes of HDAC and SIRT genes and with dasatinib sensitivity

Dasatinib is an inhibitor of multiple ABL and SRC family kinases and additional tyrosine kinases [45,67,71,72,80,83,89]. Six genes encoding its targets, *ABL2, CSK, EPHA2, MAP4K5, YES1*, and *ZAK*, were downregulated in a concerted manner by dasatinib in the NCI-TPW dataset (Supplementary Table S7). All of them were downregulated at 24 hr, and some of them were also downregulated at earlier time points after treatment. None of the remaining 16 dasatinib kinase target genes satisfied the criteria for concerted expression changes under any condition. *EPHA2* showed the strongest downregulation. It was downregulated at each time point (2, 6, and 24 hr) by both concentrations of dasatinib and had the largest amplitude of concerted downregulation after treatment with the high concentration (Figure S8; Supplementary Table S7). The second most frequently downregulated target gene was *ABL2*, with was downregulated at 6 and 24 hr by both concentrations of dasatinib (Supplementary Table S7).

Supplementary Table S8 shows Spearman correlations of expression changes and baseline expression of dasatinib kinase target genes with log(GI50) of dasatinib that satisfy *p*_FDR_ <0.1. Downregulation of *EPHA2, CSK, ZAK*, and *MAP4K5* was significantly associated with dasatinib sensitivity (0.3542 ≤ ρ  ≤0.5833; 2.97 × 10^−6^ ≤ *p*_0_ ≤0.0086; 0.0004 ≤ *p*_FDR_ ≤0.0827). Most notably, post-treatment downregulation of *EPHA2* was associated with sensitivity with *p*_FDR_ ≤0.05 at each time point and each concentration. Furthermore, the correlation of downregulation of *EPHA2* by the high concentration with dasatinib sensitivity was the strongest and the most significant (0.5434 ≤ ρ  ≤0.5833; 2.97 × 10^−6^ ≤ *p*_0_ ≤5.47 × 10^−5^; 0.0004 ≤ *p*_FDR_ ≤0.0021) among all dasatinib target genes and all conditions. Changes in *PDGFRA* expression at 24 hr, which did not follow a concerted pattern, were negatively associated with sensitivity (−0.3980 ≤ ρ  ≤ −0.3893; 0.0038 ≤ *p*_0_ ≤0.0057; 0.0454 ≤ *p*_FDR_ ≤0.0627). Sensitivity to dasatinib was also associated with increased baseline expression of *PDGFRB, ABL1, YES1*, and *SRC* (−0.3497 ≤ ρ  ≤ −0.3893; 0.0038 ≤ *p*_0_ ≤0.0057; 0.0454 ≤ *p*_FDR_ ≤0.0627). Multiple associations of additional target genes did not reach statistical significance (data not shown). Our findings were consistent with earlier reports of associations of dasatinib sensitivity with high baseline expression of *YES1* and *LYN* in ovarian and breast cell lines, and of *EPHA2* in ovarian, breast and prostate cell lines [70,90]. Elevated baseline expression of all three of these genes in the NCI-TPW dataset showed trends for association with dasatinib sensitivity; however, only association of baseline *YES1* expression reached statistical significance (= −0.4245, −0.2856, and −0.2535; *p*_0_ = 0.0010, 0.0313, and 0.0571; *p*_FDR_ = 0.0071, 0.1930, and 0.2615 for *YES1, EPHA2*, and *LYN*, respectively).

Expression of several dasatinib targets was significantly associated with transcriptional changes of *HDAC* and *SIRT* genes at 6 and 24 hr after treatment with the high concentration (Supplementary Table S9). The two strongest associations were for upregulation of *HDAC5* at 24 hr with downregulation of *EPHA2* (ρ = −0.6086; *p*_0_ = 3.49 × 10^−6^; *p*_FDR_ = 0.0049) and with elevated baseline expression of *YES1* (ρ  = 0.5841; *p*_0_ = 1.05 × 10^−5^; *p*_FDR_ = 0.0074). Upregulation of *HDAC5*, downregulation of *EPHA2*, and baseline expression of *YES1* were all significantly associated with dasatinib sensitivity (Table 2; Supplementary Tables S1 and S8), suggesting an interplay among these molecular factors. In addition to its strong association with post-treatment downregulation of *EPHA2*, upregulation of *HDAC5* at 24 hr was also strongly associated with baseline *EPHA2* expression (ρ  = 0.5841; *p*_0_ = 1.05 × 10^−5^; *p*_FDR_ = 0.0074; Supplementary Table S9). This suggests possible direct effects of EphA2 both on transcriptional response of *HDAC5* and on drug sensitivity. In addition, upregulation of *HDAC5* at 24 hr was associated with increased baseline expression of *ABL1* and with decreased baseline expression of *CSK* (ρ  = 0.4679 and −0.4617, respectively; *p*_FDR_ ≤0.0892; Supplementary Table S9).

Several additional correlations of dasatinib targets with other *HDAC* and *SIRT* genes associated with dasatinib sensitivity satisfied *p*_FDR_ ≤0.01 (Supplementary Tables S1 and S9). All such correlations were positive, with ρ  ≥0.4526. They included associations of downregulation of *CSK* and *ZAK* with downregulation of *HDAC2* at 24 hr, a correlation between log_2_FC of *YES1* at 6 hr and upregulation of *SIRT1*, and correlations of baseline expression of *ABL1* and of log_2_FC of *LCK* with upregulation of *SIRT3* at 24 hr (Table 1; Supplementary Tables S7 and S9). Downregulation of *CSK* and *ZAK* and baseline expression of *ABL1* were also associated with dasatinib sensitivity (Supplementary Table S8). These associations provide further support for potential interplay among the effects of different dasatinib targets, transcriptional response of *HDAC* and *SIRT* genes to dasatinib, and dasatinib sensitivity. Multiple weaker associations of various dasatinib targets with changes in *HDAC* and *SIRT* expression did not reach statistical significance but showed similar trends consistent with sensitivity to dasatinib (data not shown). For example, baseline expression and/or downregulation of *EPHA2* was modestly associated with upregulation of *HDAC1, SIRT1, SIRT2, SIRT3, SIRT4*, and *SIRT5* (0.3936 ≤ |ρ | ≤ 0.4460, 0.0013 ≤ *p*_0_ ≤0.0051, 0.1079 ≤ *p*_FDR_ ≤0.1863). Expression of *PDGFRA, PDGFRB, ABL2, BCR, FRK, MAPK14, CSF1R*, and other dasatinib target genes was also weakly correlated with transcriptional changes of *HDAC* and *SIRT* genes (data not shown).

## Discussion

Our study uncovered an unexpected strong significant association of *HDAC5* upregulation with sensitivity to the kinase inhibitors dasatinib and erlotinib and a weaker similar trend for lapatinib sensitivity. These associations are contrary to prior reports which found the benefits of downregulating HDAC5 in tumours and detrimental effects of HDAC5 upregulation on tumour progression. Using co-immunoprecipitation and immunoblotting of HDAC5 and SOX9, treatment with the HDAC4 and HDAC5 inhibitor LMK-235, differential gene expression analysis, *HDAC5* knockouts generated using CRISPR/Cas9, and HDAC5 overexpression using plasmid transfection, Xue et al. [86] demonstrated that HDAC5 was essential for deacetylation and nuclear localization of SOX9, which was important for resistance to tamoxifen in breast cancer cell lines. Multiple studies that used depletion, inhibition, or overexpression of HDAC5 have shown that it participates in the maintenance of pericentric heterochromatin and cell cycle control, and in regulation of cell survival, proliferation, migration, invasion, and apoptosis [8,23,91]. The roles of HDAC5 in repression of cyclin D3 (CCND3) levels and cell cycle regulation have been demonstrated using HDAC5 overexpression, depletion, and cytoplasmic sequestration experiments, by cell treatment with the HDAC class I and II inhibitor trichostatin A, and through serial deletions of the *CCND3* promoter region [91]. Using coimmunoprecipitation and deacetylation assays, and with the help of knockdown and overexpression experiments of HDAC5, Sharma et al. demonstrated the role of HDAC5 in modulation of transcriptional activity of SATB1, a genome organizer which recruits transcriptional modellers, and in promotion of aggressive features of lung adenocarcinoma in cell lines and orthotopic mouse xenograft models [92]. Elevated mRNA or protein expression of HDAC5 led to an increased proliferation and invasiveness of a lung cancer cell line and was associated with inferior prognoses and clinical outcomes in patients with breast cancer and hepatocellular carcinoma [22,23,93,94]. Depletion, inhibition, or silencing of HDAC5 in breast cancer, cervical carcinoma, and melanoma cell lines, and in hepatocellular carcinoma cell lines under hypoxia delayed cell cycle progression, reduced cell proliferation, migration, invasion and survival, induced apoptosis and autophagy and increased sensitivity to the DNA damaging agents doxorubicin and cisplatin [8,23,94,95]. Similarly, silencing of HDAC5 inhibited tumour growth in melanoma mouse xenograft models [95]. In glioma cell lines, cell proliferation was increased when HDAC5 was overexpressed and decreased with HDAC5 silencing [24]. While these reports seemingly contradict our findings of the association of *HDAC5* upregulation with sensitivity to kinase inhibitors, HDAC5 overexpression had dual effects in urothelial carcinoma cell lines, decreasing cell proliferation in multiple cell lines but increasing the epithelial-to-mesenchymal transition in one cell line [88]. The latter effect may be in agreement with the *HDAC5* associations observed in our study. It is possible that the differences in the directions of associations may be caused by different underlying interactions of HDAC5 with inhibitors of signalling pathways as opposed to the effect of HDAC5 on response to cytotoxic therapies.

Similar to *HDAC5*, the increase in *HDAC1* expression was significantly associated with sensitivity to dasatinib in our study, even though the transcriptional response of *HDAC1* to dasatinib was variable among the NCI-60 cell lines and did not satisfy the criteria for concerted upregulation (Tables 1 and 2; Figure S4). HDAC1 is involved in many important epigenetic interactions with multiple partners. It is an important factor in the regulation of chromatin remodelling, DNA methylation, transcription, and apoptosis [15,16,30,96]. While we observed an association of *HDAC1* upregulation with sensitivity to dasatinib, upregulation of *HDAC1* after treatment was associated with resistance to the DNA damaging agents topotecan, cisplatin, and gemcitabine. The latter result is consistent with the reports of increased resistance to the DNA damaging agent etoposide and increased multi-drug resistance to DNA damaging agents when *HDAC1* levels were elevated, and of the benefits of *HDAC1* silencing [2,29]. Our findings add to the body of evidence suggesting a potential benefit of downregulating *HDAC1* levels in order to improve the efficacy of DNA damaging therapy.

Upregulation of several *SIRT* genes was also associated with increased sensitivity to dasatinib. Associations of *SIRT3* and *SIRT4* were in agreement with earlier reports about their effects on tumour suppression and on improved patient outcomes in hepatocellular carcinoma, and they are consistent with associations of higher *SIRT3* expression levels with drug sensitivity in HCC cell lines [32]. By contrast, associations of dasatinib sensitivity with stronger upregulation of *SIRT1, SIRT2*, and *SIRT5* were unexpected, since these genes have tumour promoting effects, and their downregulation positively affects cancer treatment outcomes [13,14,32,78,79]. Higher *SIRT1* levels had been associated with resistance to the kinase inhibitor sorafenib and to multiple other antitumor agents including cisplatin, oxaliplatin, 5-fluorouracil, doxorubicin, and pirarubicin in a variety of tumours [32,79,97]. The direction of association of *SIRT2* expression changes in NCI-TPW contrasts with earlier reports of increased sensitivity to dasatinib in melanoma cell lines with *SIRT2* knockdown [31]. Molecular mechanisms which may explain these opposite effects of *SIRT2* on dasatinib sensitivity require further investigation. They may include, e.g., pretreatment differences between non-manipulated cancer cell lines and cell lines with *SIRT2* knockdown. *SIRT2* silencing prior to treatment broadly impacts biological processes in the cell [32], which may contribute to dasatinib response. Such pretreatment effects would be absent from the NCI-60 cell line panel profiled in the NCI-TPW dataset, which had not been genetically manipulated. While *SIRT1, SIRT2*, and *SIRT5* may have tumour-promoting or tumour-suppressing effects depending on the tumour histology and cellular context [32], the NCI-60 panel included melanoma cell lines, and therefore the differences in *SIRT2* associations with dasatinib response may be caused by factors other than tumour specificity.

Among multiple associations of *HDAC* and *SIRT* mRNA expression changes with response to dasatinib and erlotinib with *p*_FDR_ <0.1, only downregulation of *HDAC2* was associated with increased sensitivity to dasatinib (Table 2; Supplementary Table S1). In contrast, all correlations of log_2_FC of *HDAC1, HDAC5, SIRT1, SIRT2, SIRT3, SIRT4*, and *SIRT5* with log(GI50) of these agents satisfying *p*_FDR_ <0.1 were negative, indicating that their higher expression levels after treatment were associated with sensitivity to these agents. It remains to be investigated whether there is a causative effect of upregulation of these *HDAC* and *SIRT* genes on sensitivity to kinase inhibitors. In untreated cells, higher levels of *HDAC1* and *HDAC7* (Supplementary Table S2) were similarly associated with sensitivity to the kinase inhibitors sunitinib and dasatinib, respectively. In both analyses the cell lines with increased mRNA levels of *HDACs* were more sensitive to kinase inhibitors, however, the gene-agent combinations with significant associations of baseline expression were different from those with significant log_2_FC associations. Despite these differences, if the associations of sensitivity to kinase inhibitors with *HDAC* upregulation and with *HDAC* levels in untreated cells have a common mechanism, then elevation of *HDAC* and *SIRT* levels in drug-sensitive cells after treatment is unlikely be a mere biomarker of increased cell death in response to treatment and is more likely to involve other molecular mechanisms.

We observed concerted changes in mRNA expression of *HDAC* and *SIRT* genes in response to various antitumor agents in NCI-TPW, including many cases of transcriptional upregulation. Independent datasets showed patterns similar to NCI-TPW for mRNA and protein expression changes of HDACs and SIRTs in response to dasatinib and vorinostat in malignant and non-malignant cell lines. We experimentally confirmed transcriptional and proteomic upregulation of HDAC5 by dasatinib. Our findings show considerable effects of drug treatment on expression of epigenetic factors, even when using non-epigenetic agents. Surprisingly, vorinostat upregulated transcription of *HDAC1* and *HDAC3*, even though the products of both genes are targets of vorinostat inhibition. Vorinostat induces profound transcriptional changes in many genes [34,76,98,99], and potential clinical consequences of its role in upregulation of *HDAC1, HDAC3, HDAC5, SIRT2*, and other *SIRT* genes may need to be considered.

Among the associations with vorinostat response, only the amplitude of expression changes of *SIRT2* had *p*_FDR_ ≤0.1 and was associated with sensitivity to that agent (high concentration, 6 hr; ρ  = −0.4033, *p*_FDR_ = 0.0909; Supplementary Table S1). Upregulation of *SIRT2* by vorinostat was in agreement with transcriptional data for malignant and non-malignant cells from the GEO datasets GSE43010 [47] and GSE84205 [46] and the study by Kyrylenko et al. [7]. SIRT2 protein levels were also upregulated by vorinostat in fibroblasts with Newman-Pick type C1 disease [43]. Transcriptional upregulation of *SIRT2* by the HDAC inhibitor trichostatin A in neuroblastoma cell lines had been linked to hyperacetylation of the DNA-bound histone H4 in the *SIRT2* promoter region [7]. Such upregulation is an example of important influence of tumour drug treatment on *HDAC* and *SIRT* expression.

Three miRNAs, *miR-125a-5p, miR-589-5p*, and *miR-*217, which regulate HDAC5, were upregulated by vorinostat in the three AML cell lines in the GEO dataset GSE69959 (Figure S6). Upregulation of *miR-125a-5p* by vorinostat in AML cell lines is consistent with its previously reported upregulation by another HDAC inhibitor, AR42, in the ovarian cancer cell line CP70 [100].

Our transcriptome-wide enrichment analysis of NCI-TPW data found significant associations with response to the high concentration of dasatinib at 24 hr for the gene targets of the same three miRNAs, *miR-125a-5p, miR-589-5p*, and *miR-*217, which were upregulated by vorinostat in GSE69959 (Figure S6; Supplementary Table S5). The agreement between the findings from both analyses, in which the same miRNAs *miR-125a-5p, miR-589-5p*, and *miR-*217 were consistently upregulated by vorinostat and had their target genes associated with dasatinib response, may suggest potential roles of these three miRNAs in regulation of HDAC5 in response to treatment by both agents. Upregulation of *miR-125a* and *miR-125a-5p* in cancer cells by HDAC inhibitors and regulation of HDAC5 protein levels by *miR-125a-5p* was experimentally demonstrated [54,100]. It is surprising that expression of both *HDAC5* and *miR-125a-5p, miR-589-5p*, and *miR-*217 was upregulated by vorinostat, as HDAC5 and *miR-125a-5p* were reported to negatively affect each other’s levels [54,101]. Similarly, *miR-589-5p* negatively affects HDAC5 levels, and upregulation of *mir-589-5p* in tumour cells by 5-aza-2-deoxycytidine downregulated HDAC5 [52]. Concurrent upregulation of *miR-125a-5p, miR-589-5p*, and HDAC5 by vorinostat may involve additional molecular factors which remain to be identified.

*miR-9* and *miR-124* had a mixed response to vorinostat, as they were upregulated by vorinostat only in a subset of the AML cell lines in GSE69959. Their target genes were not associated with response to dasatinib in NCI-TPW. Both miRNAs regulate HDAC5 levels in non-malignant neural cells [53,57], and *miR-9* is also involved in HDAC5 regulation in Waldenström macroglobulinemia, a B-cell low-grade lymphoma. It is possible that miR-9 and miR-124 may not directly regulate HDAC5 levels in response to vorinostat and dasatinib.

In addition to miRNA-mediated regulation, HDAC levels and activity are affected by multiple other post-transcriptional and post-translational regulatory mechanisms, including their interactions with other proteins and a variety of protein modifications [1], some of which could affect HDAC5 after treatment with dasatinib and vorinostat. While our study examined changes in mRNA and protein levels of HDACs and SIRTs, future studies may investigate how drug treatment may affect their post-translational modifications, and the potential effects of chemotherapy on their subcellular localization, as individual HDACs and SIRTs have different distributions in cellular compartments, and some of them are dynamically shuttled between the nucleus and the cytosol [42,59,102–104].

Our analysis showed that dasatinib induced transcriptional downregulation of its several kinase target genes (Supplementary Table S7). Our results revealed interdependence among expression of several dasatinib kinase targets, changes in expression of multiple *HDAC* and *SIRT* genes induced by dasatinib treatment, and sensitivity to that agent. The strongest associations were between downregulation of *EPHA2* by dasatinib, upregulation of *HDAC5*, and dasatinib sensitivity. EphA2 (Ephrin Type A-Receptor 2), the product of *EPHA2*, is an important tyrosine kinase receptor target of dasatinib. It is frequently overexpressed in malignant cells and has been suggested to be an important factor in tumour growth, survival, metastatic processes, and angiogenesis [69,105] Our findings add to the body of evidence [70,90] suggesting the influence of EphA2 on tumour response to dasatinib. Our results further indicate that these effects of EphA2 may be mediated by HDAC5, or that *HDAC5* expression may be a biomarker of such effects. Our findings underscore the complexity of interactions among the effects of dasatinib on its multiple protein kinase targets and on transcriptional regulation of *HDAC* and *SIRT* genes. Interestingly, Karwaciak et al. [31] demonstrated that downregulation of *SIRT2* in melanoma cell lines sensitized them to dasatinib, decreased mRNA and protein expression of EphA2, and altered mRNA expression of several other kinase target genes of dasatinib. In the NCI-TPW dataset, dasatinib sensitivity was significantly associated with the increase in *SIRT2* expression and with downregulation of *EPHA2* after treatment (Tables 1 and 2; Supplementary Tables S1, S7 and S8). At 24 hr after treatment with the high concentration, *SIRT2* and *EPHA2* expression changes were negatively correlated with each other (ρ  = 0.4051, *p*_0_ = 0.0039; *p*_FDR_ = 0.1610). Of note, elevated baseline expression of *EPHA2* and of several other dasatinib kinase target genes, e.g., *ABL1, PDGRFB, YES1*, and *SRC*, was associated with dasatinib sensitivity and was positively correlated with upregulation of both *HDAC5* and *SIRT2* at 24 hr (e.g., ρ  = 0.5841 and 0.3570, *p*_0_ = 1.05 × 10^−5^ and 0.0118 for the correlations of baseline *YES1* expression with upregulation of *HDAC5* and *SIRT2*, respectively). Such pretreatment associations of dasatinib targets suggest that EphA2, YES1, and other targets may have causative influences on transcriptional regulation of *HDAC* and *SIRT* genes in response to treatment and on dasatinib sensitivity. However, the findings of Karwaciak et al. [31] also suggest the presence of a feedback loop, whereby changes in the expression of *SIRT2*, and possibly of other *HDAC* or *SIRT* genes, may modulate the levels and activity of dasatinib kinase targets.

Xue et al. [86] reported transcriptional upregulation of *HDAC5* by *c*-Myc in tamoxifen-resistant breast cancer cells and suggested its role in resistance to tamoxifen. In NCI-TPW, after treatment with both dasatinib and vorinostat, the expression of *MYC* was decreased in many cell lines, including MCF-7 and T47D studied by Xue et al. [86] (Figure S9). Dasatinib-induced downregulation of *MYC* at 2, 6, and 24 hr at both high and low concentrations satisfied the criteria for concerted changes [33,35]. Downregulation of *MYC* suggests that transcriptional upregulation of *HDAC5* by both agents and its potential effect on sensitivity to dasatinib may be mediated by factors other than *c*-Myc.

Studies of NSCLC cell lines and patient samples noted the effect of inactivating BRAF mutations Y472C and G466V on increased sensitivity to dasatinib [45,71]. None of the NCI-60 cell lines have been reported to carry these inactivating mutations [49]. In contrast, several NCI-60 cell lines carry the activating mutation BRAF V600E. Among them are the colorectal cell lines COLO-205 and HT29, and multiple melanoma cell lines [49,106]. Both colorectal cell lines and the melanoma cell line LOX were sensitive to dasatinib (log(GI50) between −7.908 and −7.657 in NCI-TPW), whereas many other melanoma cell lines with BRAF V600E were resistant to dasatinib (log(GI50) close to −5; Figure S5). It is therefore unlikely that activating or inactivating mutations in *BRAF* may be involved in the mechanism of association of upregulation of *HDAC1, HDAC5*, and *SIRT*s with dasatinib sensitivity.

Since upregulation of *HDAC1, HDAC5*, and multiple *SIRT*s was associated with sensitivity to dasatinib, and *HDAC1, HDAC5, SIRT2, SIRT3*, and *SIRT4* were upregulated by vorinostat (Tables 1 and 2), it would be reasonable to investigate whether a combination of these agents may have a synergistic antitumor activity. To date, the phase 1 clinical trial NCT00816283 has been completed after testing this combination in patients with accelerated phase or blastic phase CML or ALL, however the trial data have not been posted [107–109]. *In vitro* studies found synergy between vorinostat and dasatinib in pancreatic and SCLC cell lines and in SCLC cell line-derived xenografts [66,110]. In contrast, the NCI ALMANAC study, a comprehensive study of drug combinations which used the NCI-60 cancer panel [48], found the synergy between dasatinib and vorinostat to be limited to a very small number of cell lines. The NCI-60 panel does not include either SCLC or pancreatic cell lines. Two independent studies found synergistic antitumor effects of dasatinib and vorinostat in two cell lines included in the NCI-60 panel. Among them, the CML cell line K-562 [111] also showed synergy of that combination in the NCI-ALMANAC (ComboScore = 110) [48]. In contrast, the synergy of vorinostat and dasatinib reported for the breast cancer cell line MCF-7 in a separate study [112] was not observed in the NCI-ALMANAC (Combo Score = −93). Combi-nation studies of other HDAC inhibitors observed inhibition of the CML K-562 cell line by a combination of dasatinib with the class I HDAC inhibitor MS-275 (entinostat) [113], and of thyroid cancer cell lines with dasatinib combinations with pan-histone inhibitors belinostat or panobinostat [114].

Molecular interactions between dasatinib and vorinostat or other HDAC inhibitors are complex and involve multiple pathways and targets [66,110–112,115]. Vorinostat enzymatically inhibits several HDACs and induces broad transcriptional changes in multiple genes [25,74–77]. The multi-kinase inhibitor dasatinib induces a variety of antitumor effects through a broad range of biological processes, including apoptosis, autophagy, necroptosis, cell cycle arrest, and immune modulation, and the response to this agent has been associated with multiple biomarkers [45,67,71,80,83,89,116]. Interactions between dasatinib and vorinostat may involve additional mechanisms beyond transcriptional upregulation of *HDAC* and *SIRT* genes. Examples of such mechanisms were suggested by Mahendrarajah et al. [113] who attributed the synergy between MS-275 and dasatinib in the K-562 cell line to inactivation of p-ACK1 and p-STAT3, and by Chan et al. [114] who noted that thyroid cancer cell lines sensitive to dasatinib combinations with HDAC inhibitors carried mutations in the components of the MAPK pathway. Yang et al. [66] suggested that YAP1 and other components of Hippo signalling contribute to synergy between HDAC inhibitors and dasatinib sensitivity. We observed weak, non-statistically significant trends for associations of *YAP1* expression with the NCI-60 cell line responses to vorinostat and dasatinib, in the same directions as those reported by Yang et al. [66] However, while expression changes of many *HDAC* and *SIRT* genes in the NCI-TPW dataset were strongly and significantly associated with dasatinib sensitivity (Table 2; Supplementary Table S1), they had only a weak or no association with *YAP1* expression (Supplementary Table S6). This suggests that YAP1 may not be the main driver of the biological links between transcriptional changes of *HDAC* and *SIRT* genes and dasatinib sensitivity. In addition, the majority of the studies of combinations of HDAC inhibitors and dasatinib *in vitro* used simultaneous treatment with both inhibitors. In order to investigate whether upregulation of the genes encoding histone deacetylases enhances the potency of dasatinib, future drug combination studies of HDAC inhibitors and dasatinib may benefit from a sequential drug treatment, providing a sufficient time for transcriptional upregulation between treatments by different agents. If conducted in specific tumour categories, such analysis could avoid potential differences among tumour histologies in drug response and in *HDAC* and *SIRT* expression.

Our study provides a novel summary of the effects of 15 antitumor agents on transcriptional changes of *HDACs* and *SIRTs*. Additionally, our investigation of associations of drug response with baseline expression and expression changes of *HDAC* and *SIRT* genes revealed previously unknown associations between upregulation of several *HDAC* and *SIRT* genes and sensitivity of cancer cell lines to kinase inhibitors. Further investigation may reveal whether such upregulation has a causal effect or is a biomarker of sensitive cells responding to treatment. If these associations are causative, they may need to be considered during chemotherapy treatment.

## Conclusions

Our analysis revealed concerted upregulation and downregulation of multiple *HDAC* and *SIRT* genes in response to cell line treatment with approved chemotherapy agents. The amplitude of transcriptional upregulation of *HDAC5, HDAC1*, and several *SIRT* genes after drug treatment was associated with sensitivity to kinase inhibitors. The strongest associations were observed with sensitivity to dasatinib. The transcriptional effect of cancer drug treatment on genes encoding histone deacetylases may be worthy of further investigation for potential clinical implications.

## Supplementary Material

Supplemental Material

## Data Availability

The datasets analysed in the current study are publicly available from the NCI-TPW portal, [https://tpwb.nci.nih.gov] [33] (NCI-TPW drug response data, gene expression changes, and baseline gene expression data and their graphical presentation) and NCBI GEO, [https://www.ncbi.nlm.nih.gov/geo/] (public datasets used for validation of *HDAC* and *SIRT* and miRNA expression changes in response to drug treatment). The dataset of mRNA and protein changes of HDAC5 in the IGROV1, MDA-MB-231, and UACC-257 cell lines in response to dasatinib used during the current study is available from the corresponding author on reasonable request.
