# Supplementary material for: Association of changes in expression of HDAC and SIRT genes after drug treatment with cancer cell line sensitivity to kinase inhibitors
Source: Epigenetics. 2024 Feb 18;19(1):2309824. doi: 10.1080/15592294.2024.2309824 (PMC10878021; doi:10.1080/15592294.2024.2309824)
Supplement: Supplemental Material [file KEPI_A_2309824_SM1624.zip › Supplementary figure and table legends.docx]

**Supplementary Material**

**Supplementary Figures**

**Figure S1. The workflow representing the computational and experimental steps of the study and sources of data**

The initial computational discovery analysis using NCI-TPW data identified concerted expression in *HDAC* and *SIRT* genes and used Spearman and Pearson correlation analyses to identify those genes whose expression changes (**log_2_FC)** or baseline expression were associated with drug response. Validation of the initial findings included experimental analysis using RT-PCR and Western blots and computational analysis using publicly available data from the NCBI GEO (National Center for Biotechnology Information Gene Expression Omnibus) and biomedical publications. Additional computational analyses of miRNAs involved in HDAC5 regulation used publicly available data from NCI-TPW and NCBI GEO.

**NCI-TPW**, The NCI Transcriptional Pharmacodynamics Workbench. **GSE51083, GSE69395, GSE84205** **GSE43010**, and **GSE69959** were public datasets obtained from NCBI GEO.

**Figure S2. Changes in expression of *HDAC4* and *HDAC9* in the NCI-60 cell line panel in response to treatment with bortezomib and gemcitabine**

Shown are transcriptional changes (log_2_FC) at 2 (left panel), 6 (middle panel), and 24 hr (right panel) after treatment. Horizontal right bars indicate elevated gene expression, whereas left bars decreased expression relative to untreated cell lines. **(A)** *HDAC4* after treatment with the high concentration of bortezomib (100 nM). **(B)** *HDAC4* after treatment with the low concentration of bortezomib (10 nM). **(C)** *HDAC9* after treatment with the low concentration of bortezomib. **(D)** *HDAC9* after treatment with the high concentration of gemcitabine (2000 nM). Concerted expression changes for each of these genes and conditions were observed at 24 hr, as shown on the right most panels. Colors represent cancer categories (breast, central nervous system (CNS), colon, leukemia, lung, melanoma, ovarian, prostate, and renal cancers), with the legend provided at the top of the figure. The scale on the bottom represents log_2_ difference between expression values of treated and untreated cell lines. The scale for each microarray experiment is specific to that experiment.

**Figure S3. Examples of expression changes of *HDAC* and *SIRT* genes in the NCI-60 cell line panel in response to treatment with the high concentration of vorinostat**

Shown are transcriptional changes (log_2_FC) at 2 (left panel), 6 (middle panel), and 24 hr (right panel) after treatment with the high concentration of vorinostat (5000 nM). Horizontal right bars indicate elevated gene expression, whereas left bars indicated decreased expression relative to the untreated cell lines. **(A)** *HDAC1* (upregulated); **(B)** *HDAC5* (upregulated); **(C)** *HDAC7* (downregulated); **(D)** *SIRT4* (upregulated). For each of these genes and conditions, concerted expression changes were observed at 24 hr, as shown on the right most panels. Colors represent cancer categories (breast, central nervous system (CNS), colon, leukemia, lung, melanoma, ovarian, prostate, and renal cancers), with the legend provided at the top of the figure. The scale on the bottom represents log_2_ difference between expression values of treated and untreated cell lines. The scale for each microarray experiment is specific to that experiment.

**Figure S4. Changes in expression of *HDAC* and *SIRT* genes in response to treatment with dasatinib and erlotinib**

Shown are transcriptional changes (log_2_FC) at 2 (left panel), 6 (middle panel), and 24 hr (right panel) after treatment. Horizontal right bars indicate elevated gene expression, whereas left bars decreased expression relative to untreated cell lines. Expression of *HDAC1* after treatment with **(A)** the high concentration (2000 nM) of dasatinib or **(B)** the high concentration (10000 nM) of erlotinib did not satisfy the criteria for concerted expression changes at any time point. **(C)** *SIRT3* expression after treatment with the high concentration of dasatinib, showing concerted upregulation at 24 hr (right panel). **(D)** Expression of *SIRT5* after treatment with the high concentration of erlotinib, with concerted upregulation observed at 24 hr (right panel). **(E)** *HDAC7* expression after treatment with the high concentration of dasatinib showed concerted downregulation at 24 hr (right panel). **(F)** *SIRT2* expression after treatment with the low concentration (100 nM) of dasatinib showed concerted upregulation at 24 hr (right panel). Colors represent cancer categories. The scale on the bottom represents log_2_ difference between expression values of treated and untreated cell lines. The scale for each microarray experiment is specific to that experiment.

**Figure S5. Scatterplots of transcriptional changes (log_2_FC) in *HDAC5* expression at 24 hr after treatment with dasatinib vs log(GI50) of the NCI-60 cell lines**

**(A)** High (2,000 nM) concentration of dasatinib. **(B)** Low (100 nM) concentration of dasatinib. Colors represent cancer categories, as shown in the color legend. Horizontal axis provides log(GI50) values. Vertical axis represents provides log_2_FC values (difference in gene expression between treated and untreated cell lines, provided as log_2_, in treated cells relative to baseline values). **ρ**, Spearman correlation coefficient. ***r****,* Pearson correlation coefficient. ***p*_FDR_**, FDR adjusted *p*-values for Spearman and Pearson correlation analyses.

**Figure S6. Vorinostat-induced changes in normalized expression values of miRNAs with reported regulatory effects on HDAC5, based on the data from the GEO dataset GSE69959**

Shown are the log_2_-transformed normalized changes in expression of the miRNA in the treated vs untreated cell lines values at 6 hr after treatment with 5 μM of vorinostat as compared to the pretreatment levels using publicly available data from GSE69959 ^59^. The data are for the leukemia cell lines KASUMI1, U937, and K562. Expression changes were averaged among the 5 probes and 3 technical replicates for each miRNA and each cell line. miRNA names in the dataset GSE69959 were, **miR-125a-5p:** hsa-miR-125a-5p; **miR-589-5p:** hsa-miR-589; **miR-217:** hsa-miR-217; **miR-124:** hsa-miR-124; **miR-9:** hsa-miR-9

**Figure S7. Changes in *YAP1* expression in response to treatment with the high concentration of dasatinib and both concentrations of vorinostat**

Shown are transcriptional changes (log_2_FC) of *YAP1* at 2 hr (left panel), 6 hr (middle panel), and 24 hr (right panel) after treatment, as compared to untreated cells. Horizontal right bars indicate elevated gene expression, whereas left bars show decreased expression relative to untreated cell lines. Changes in *YAP1* expression are shown after treatment with **(A)** the high concentration (2000 nM) of dasatinib; **(B)** the low concentration (1000 nM) of vorinostat; and **(C)** the high concentration (5000 nM) of vorinostat. Colors represent cancer categories. Transcriptional downregulation of *YAP1* at 6 and 24 hr satisfied criteria for concerted changes for all three of these conditions. The scale on the bottom represents log_2_ difference between expression values of treated and untreated cell lines. The scale for each microarray experiment is specific to that experiment.

**Figure S8. Downregulation of *EPHA2* by dasatinib**

Shown are transcriptional changes (log_2_FC) of *EPHA2* at 2 hr (left panel), 6 hr (middle panel), and 24 hr (right panel) after treatment, as compared to untreated cells. Horizontal right bars indicate elevated gene expression, whereas left bars show decreased expression relative to untreated cell lines. Changes in *EPHA2* mRNA expression are shown after treatment with **(A)** the high (2000 nM) and **(B)** the low (100 nM) concentrations of dasatinib. Transcriptional downregulation of *EPHA2* at each of the three time points and both concentrations of dasatinib satisfied the criteria for concerted changes (Supplementary Table S7). The scale on the bottom represents log_2_ difference between expression values of treated and untreated cell lines. The scale for each microarray experiment is specific to that experiment.

**Figure S9. Changes in *MYC* expression in response to treatment with the high concentrations of dasatinib and vorinostat**

Shown are transcriptional changes (log_2_FC) of *MYC* at 2 (left panel), 6 (middle panel), and 24 hr (right panel) after treatment, as compared to untreated cells. Horizontal right bars indicate elevated gene expression, whereas left bars decreased expression relative to untreated cell lines. Changes in *MYC* expression are shown after treatment with **(A)** the high concentration (2000 nM) of dasatinib and **(B)** the high concentration (5000 nM) of vorinostat. Colors represent cancer categories. The scale on the bottom represents log_2_ difference between expression values of treated and untreated cell lines. The scale for each microarray experiment is specific to that experiment.

**Supplementary Tables**

**Supplementary Table S1. Spearman correlations between log(GI50) and log_2_FC of *HDAC* and *SIRT* genes satisfying *p*_FDR_ < 0.1**

Shown are the Spearman correlations satisfying *p*_FDR_ < 0.1. Correlations satisfying a more stringent criterion of *p*_FDR_ < 0.05 are marked with an asterisk (*). Colors highlight the associations with the kinase inhibitors dasatinib and erlotinib. **ρ,** Spearman correlation coefficient. **Condition** indicates concentration (**high** or **low**) and the time point (**2**, **6**, or **24** hr after treatment). ***p*_0_,** *p*-value prior to FDR adjustment; ***p*_FDR_,** FDR adjusted *p*-value.

**Supplementary Table S2. Spearman correlations of baseline expression of *HDAC* and *SIRT* genes with drug response satisfying *p*_FDR_ < 0.1**

Shown are the Spearman correlations satisfying *p*_FDR_ < 0.1. Correlations satisfying a more stringent criterion of *p*_FDR_ < 0.05 are marked with an asterisk (*). Colors highlight negative (yellow) and positive (green) associations with response to kinase inhibitors. **ρ,** Spearman correlation coefficient; ***p*_0_,** *p*-value prior to FDR adjustment; ***p*_FDR_,** FDR adjusted *p*-value.

**Supplementary Table S3. Comparison of transcriptional changes in cancer cell lines in response to dasatinib treatment in public NCBI GEO datasets to changes after dasatinib treatment in NCI-TPW**

**A. Comparison of changes in mRNA expression in the K-562 cell line in the NCBI GEO dataset GSE51083 to concerted changes in NCI-TPW at 24 hr after treatment with dasatinib**

Shown is the comparison of the direction of transcriptional changes in the K-562 cell line at 24 hr after treatment with 100 nM of dasatinib (after averaging the log_2_FC values among the multiple probes for each transcript and among the three replicate measurements) in GSE51083 [1] to the direction of consensus transcriptional changes in the NCI-TPW dataset at 24 hr after dasatinib treatment. **log_2_FC at 24 hr** indicates the changes in expression in GSE51083; for those changes satisfying|log_2_FC| > 0.1, positive values (upregulation after treatment) are shown in red, and negative values (downregulation after treatment) are shown in blue.

**Y** (highlighted in yellow) indicates an agreement between the direction of transcriptional changes in GSE51083 and the direction of concerted changes in NCI-TPW, for those genes with |log_2_FC| > 0.1 in GSE51083, which also had concerted expression changes at 24 hr after treatment in NCI-TPW, and had the same direction of transcriptional change (both positive or both negative log_2_FC) in both datasets. **|log_2_FC| ≤ 0.1** indicates a small change in the GSE51083 dataset; such genes were excluded from comparisons. The genes with **no concerted change in NCI-TPW** or **no data in NCI-TPW** were also excluded from comparisons.

*The direction of transcriptional changes was the same in GSE51083 and NCI-TPW for each HDAC and SIRT gene satisfying both criteria (|log2FC| > 0.1 in GSE51083 and concerted changes in NCI-TPW).*

For *HDAC7,* concerted expression changes in NCI-TPW at 24 hr were observed only at the high concentration (2000 nM) of dasatinib, and the comparison was made to that condition. For all other genes satisfying concerted expression changes at 24 hr, the comparison of expression changes was made to the low concentration (100 nM) of dasatinib in NCI-TPW, which was the same concentration as that used in GSE51083 experiments.

**B. Comparison of transcriptional changes in NSCLC cell lines in the NCBI GEO dataset GSE69395 at 72 hr after treatment with dasatinib** **to concerted changes in NCI-TPW at 24 hr after treatment with dasatinib**

Shown is the comparison of the direction of transcriptional changes in four non-small cell lung cancer (NSCLC) cell lines at 72 hr after treatment with 150 nM of dasatinib in GSE69395 [2] (after averaging the log_2_FC values among the multiple probes for each transcript) to the direction of consensus transcriptional changes in the NCI-TPW dataset. For changes satisfying |log_2_FC| > 0.1, positive values (upregulation after treatment) are shown in red, and negative values (downregulation) are shown in blue.

**Number of cell lines in agreement with NCI-TPW 24 hr consensus** (highlighted in yellow) shows the number of cell lines in GSE69395 which had the same direction of transcriptional changes with the direction of concerted changes of the consensus in NCI-TPW, for the genes and cell lines with |log_2_FC| > 0.1 in GSE69395 and concerted expression changes at 24 hr after treatment in NCI-TPW. **|log_2_FC| ≤ 0.1 in all cell lines in GSE69395** indicates genes with small transcriptional changes in GSE69395; such genes were excluded from comparisons. The genes with **no concerted change in NCI-TPW** or **no data in NCI-TPW** were also excluded from comparisons.

**Supplementary Table S4. Comparison of transcriptional changes in cancer cell lines in response to vorinostat treatment in public NCBI GEO datasets to changes after vorinostat treatment in NCI-TPW**

**A. Comparison of changes in mRNA expression of non-transformed and transformed fibroblasts in the NCBI GEO dataset GSE43010** **to concerted changes in NCI-TPW at 24 hr after treatment with vorinostat**

Shown is the comparison of the direction of transcriptional changes in NCBI GEO dataset GSE43010 [1] at 24h hr after treatment with 25 μM of vorinostat, to the direction of consensus transcriptional changes in the NCI-TPW dataset at 24 hr after vorinostat treatment (if concerted transcriptional changes were observed at either high or low concentration for that gene). The dataset GSE43010 included data on BJ (normal fibroblasts) and BJ LTSTERas (transformed fibroblasts using encoding the SV40 large T and small t antigens, hTERT and H-RAS). **log_2_FC at 24 hr** indicates the changes in expression in GSE43010, after averaging the log2FC values among the multiple probes for each transcript and among the three biological replicate measurements. For the values satisfying |log_2_FC| > 0.1 in GSE43010, positive log_2_FC values indicating upregulation after treatment are shown in red, and negative values indicating downregulation are shown in blue.

**Y** (highlighted in yellow) indicates an agreement between the direction of transcriptional changes in the GSE43010 dataset and the direction of concerted changes in NCI-TPW, for those genes with |log_2_FC| > 0.1 in GSE43010, which also satisfied the condition of concerted expression changes at 24 hr after treatment in NCI-TPW, and had the same direction of transcriptional change (both positive or both negative log_2_FC) in both datasets. **|log_2_FC| ≤ 0.1** indicates a small change in expression in the GSE43010 dataset; such genes were excluded from comparisons. The genes with **no concerted change in NCI-TPW** or **no data in NCI-TPW** were also excluded from comparisons.

**B. Comparison of transcriptional changes in the MPNST cell line 90-8TL in the NCBI GEO dataset GSE84205** **to concerted changes in NCI-TPW at 24 hr after treatment with vorinostat**

Shown is the comparison of the direction of transcriptional changes in the MPNST cell line 90-8TL at 24 hr after treatment with 2 μM of vorinostat in GSE84205 [2] to the direction of consensus transcriptional changes in the NCI-TPW dataset. For the values satisfying| log_2_FC| > 0.1 in GSE84205, positive values (upregulation after treatment) are shown in red, whereas negative values (downregulation) are shown in blue.

**Y** (highlighted in yellow) indicates an agreement between the direction of transcriptional changes in the GSE84205 dataset and the direction of concerted changes in NCI-TPW, for those genes with |log_2_FC| > 0.1 in GSE84205, which also satisfied the condition of concerted expression changes at 24 hr after treatment in NCI-TPW, and had the same direction of transcriptional change (both positive or both negative log_2_FC) in both datasets. **|log_2_FC| ≤ 0.1** indicates a small change in expression in the GSE84205 dataset; such genes were excluded from comparisons. The genes with **no concerted change in NCI-TPW** or **no data in NCI-TPW** were also excluded from comparisons.

**Supplementary Table S5. Results of miRNA-targeted gene set enrichment analysis associated with response to the high concentration of dasatinib at 24 hr, for the miRNAs which regulate HDAC5 and satisfied permutation *p*-value < 0.1**

Shown are the results of the analysis of the target gene lists for *miR-125a-5p, miR-589-5p, miR-2861, miR-9, miR-124,* and *miR-217* which had the permutation p-value < 0.1 in one or both analyses (LS and/or KS). Results satisfying a more stringent criterion of permutation p < 0.05 are shown in red. hsa-***miR-125a-5p****: miR-125a-5p;* ***hsa-miR-589-5p****: miR-589-5p;* ***hsa-miR-217:*** *miR-217;* ***hsa-miR-2861:*** *miR-2861*

**Supplementary Table S6. Spearman correlations of *YAP1* expression with expression changes of *HDAC* and *SIRT* genes at 6 or 24 hr after treatment with the high concentration of dasatinib, satisfying |ρ| > 0.2 and p_0_ < 0.1**

**ρ,** Spearman correlation coefficient. ***p*_0_,** p-value prior to the FDR adjustment. Such *p-*values < 0.05 are shown in read. ***p*_FDR_,** FDR adjusted *p*-value. None of the associations were significant after the FDR adjustment. Shown are the Spearman correlations satisfying *p*_0_ < 0.1. All such associations had Spearman |ρ| > 0.2. The results are sorted by *p*_0_.

**Comparison** indicates the time point (6 or 24 hr after treatment with the high concentration of dasatinib),

and YAP1 expression measure at that time point used in comparisons: **Baseline**, median YAP1 expression in untreated cell lines, computed across 15 agents for each cell line; **log_2_FC**, changes in gene expression induced by treatment.

Correlations with those *HDAC* and *SIRT* genes whose changes in expression were associated with response to dasatinib (Supplementary Table S1) are highlighted.

**Supplementary Table S7. Dasatinib target kinase genes which showed concerted downregulation by dasatinib in the NCI-TPW dataset**

Listed are the genes encoding dasatinib targets with kinase activity which satisfied the criteria for concerted changes after treatment by dasatinib in the NCI-TPW dataset. All of them were downregulated by dasatinib.

None of the other dasatinib kinase target genes analyzed (***ABL1, BCR, BTK, CSF1R, EPHA5, EPHB4, FGR, FRK, FYN, KIT, LCK, LYN, PDGFRA, PDGFRB, MAPK14,*** and ***SRC***) satisfied the criteria for concerted changes after treatment with dasatinib under any condition.

Downregulation (**↓**) is shown for microarray experiments in which nearly all cell lines were downregulated, and no more than 15 cell lines were upregulated. Expression changes are shown for the high (**H**; 2000 nM) or low (**L**; 100 nM) concentrations of dasatinib. The time when the change was observed is also indicated. Concerted changes for multiple conditions are separated by commas.

***** Concerted downregulation as described above and the difference of log_2_ expression values between treated and untreated cells log_2_FC ≤ -1 in some cell lines

** Concerted downregulation and log_2_FC ≤ -2.5 in some cell lines

For example, **H24↓*** for *ZAK* indicates that it had a concerted downregulation at 24 hr after treatment with the high concentration of dasatinib, with log_2_ expression changes in at least some cell lines ≤ -1

**Supplementary Table S8. Spearman correlations between log(GI50) of dasatinib and log_2_FC**

**or baseline expression of its kinase target genes satisfying *p*_FDR_ < 0.1**

Shown are the Spearman correlations satisfying *p*_FDR_ < 0.1. Correlations satisfying a more stringent criterion of *p*_FDR_ < 0.05 are marked with an asterisk (*****). The results are sorted by *p*_FDR_.

**ρ**, Spearman correlation coefficient. **Condition** indicates dasatinib concentration (**High** or **Low**) and the time point (**2**, **6**, or **24** hr after treatment) for associations of log_2_FC, or **Baseline** for associations of log(GI50) with baseline expression. ***p*_0_,** p-value prior to the FDR adjustment. ***p*_FDR_,** FDR adjusted *p*-value.

Associations for *EPHA2* are shown in red font.

**Supplementary Table S9. Spearman correlations of expression of dasatinib kinase target genes with expression changes of *HDAC* and *SIRT* genes at 6 or 24 hr after treatment with the high concentration of dasatinib, which satisfied *p*_FDR_ < 0.1**

Listed are the Spearman correlations satisfying *p*_FDR_ < 0.1.

Correlations satisfying a more stringent criterion of *p*_FDR_ < 0.05 are marked with an asterisk (*).

**ρ,** Spearman correlation coefficient. ***p*_0_,** *p*-value prior to the FDR adjustment. ***p*_FDR_,** FDR adjusted *p*-value

Comparison indicates the time point (6 or 24 hr after treatment with the high concentration of dasatinib),

and expression measure of a dasatinib target gene at that time point used in comparisons:

**Baseline,** median expression of a dasatinib target gene in untreated cell lines, computed across 15 agents for each cell line; **log_2_FC,** changes in gene expression induced by treatment

Correlations with *EPHA2* expression are shown in red font.

Correlations with *HDAC5* expression are highlighted in yellow.

Correlations with additional *HDAC* and *SIRT* genes whose changes in expression were associated with response to dasatinib (Supplementary Table S1) are highlighted in green.
