# Supplementary material for: Association of changes in expression of HDAC and SIRT genes after drug treatment with cancer cell line sensitivity to kinase inhibitors
Source: Epigenetics. 2024 Feb 18;19(1):2309824. doi: 10.1080/15592294.2024.2309824 (PMC10878021; doi:10.1080/15592294.2024.2309824)
Supplement: Supplemental Material [file KEPI_A_2309824_SM1624.zip › Supplementary figures.docx]

**Supplementary Figures**

**Association of Changes in Expression of *HDAC* and *SIRT* Genes after Drug Treatment with Cancer Cell Line Sensitivity to Kinase Inhibitors**

Julia Krushkal^1^*, Yingdong Zhao^1^, Kyle Roney^2^, Weimin Zhu^3^, Alan Brooks^3^, Deborah Wilsker^3^,

Ralph E. Parchment^3,^ , Lisa M. McShane^1^, and James H. Doroshow^4^

^1^ Biometric Research Program, Division of Cancer Treatment and Diagnosis, National Cancer Institute, 9609 Medical Center Dr., Rockville, MD 20850, USA

^2^ Department of Biostatistics and Bioinformatics, George Washington University, 950 New Hampshire Ave, NW 5th Floor, Washington, DC, USA

^3^ Clinical Pharmacodynamic Biomarkers Program, Applied/Developmental Research Directorate, Frederick National Laboratory for Cancer Research, Frederick, MD, USA

^4^Division of Cancer Treatment and Diagnosis and Center for Cancer Research, National Cancer Institute, Bethesda, MD, USA

*Corresponding author. E-mail: [julia.krushkal@nih.gov](mailto:julia.krushkal@nih.gov)

**Figure S1. The workflow representing the computational and experimental steps of the study and sources of data**

The initial computational discovery analysis using NCI-TPW data identified concerted expression in *HDAC* and *SIRT* genes and used Spearman and Pearson correlation analyses to identify those genes whose expression changes (**log_2_FC)** or baseline expression were associated with drug response. Validation of the initial findings included experimental analysis using RT-PCR and Western blots and computational analysis using publicly available data from the NCBI GEO (National Center for Biotechnology Information Gene Expression Omnibus) and biomedical publications. Additional computational analyses of miRNAs involved in HDAC5 regulation used publicly available data from NCI-TPW and NCBI GEO.

**NCI-TPW**, The NCI Transcriptional Pharmacodynamics Workbench. **GSE51083, GSE69395, GSE84205** **GSE43010**, and **GSE69959** were public datasets obtained from NCBI GEO.

**Figure S2. Changes in expression of *HDAC4* and *HDAC9* in the NCI-60 cell line panel in response to treatment with bortezomib and gemcitabine**

Shown are transcriptional changes (log_2_FC) at 2 (left panel), 6 (middle panel), and 24 hr (right panel) after treatment. Horizontal right bars indicate elevated gene expression, whereas left bars show decreased expression relative to untreated cell lines.

**(A)** *HDAC4* after treatment with the high concentration of bortezomib (100 nM). **(B)** *HDAC4* after treatment with the low concentration of bortezomib (10 nM). **(C)** *HDAC9* after treatment with the low concentration of bortezomib. **(D)** *HDAC9* after treatment with the high concentration of gemcitabine (2000 nM).

Concerted expression changes for each of these genes and conditions were observed at 24 hr, as shown on the right most panels. Colors represent cancer categories (breast, central nervous system (CNS), colon, leukemia, lung, melanoma, ovarian, prostate, and renal cancers), with the legend provided at the top of the figure. The scale on the bottom represents log_2_ difference between expression values of treated and untreated cell lines. The scale for each microarray experiment is specific to that experiment.

**Figure S2**

**Figure S3. Examples of expression changes of *HDAC* and *SIRT* genes in the NCI-60 cell line panel in response to treatment with the high concentration of vorinostat**

Shown are transcriptional changes (log_2_FC) at 2 (left panel), 6 (middle panel), and 24 hr (right panel) after treatment with the high concentration of vorinostat (5000 nM). Horizontal right bars indicate elevated gene expression, whereas left bars indicated decreased expression relative to the untreated cell lines.

**(A)** *HDAC1* (upregulated); **(B)** *HDAC5* (upregulated); **(C)** *HDAC7* (downregulated); **(D)** *SIRT4* (upregulated). For each of these genes and conditions, concerted expression changes were observed at 24 hr, as shown on the right most panels.

Colors represent cancer categories (breast, central nervous system (CNS), colon, leukemia, lung, melanoma, ovarian, prostate, and renal cancers), with the legend provided at the top of the figure. The scale on the bottom represents log_2_ difference between expression values of treated and untreated cell lines. The scale for each microarray experiment is specific to that experiment.

**Figure S3**

**Figure S4. Changes in expression of *HDAC* and *SIRT* genes in response to treatment with dasatinib and erlotinib**

Shown are transcriptional changes (log_2_FC) at 2 (left panel), 6 (middle panel), and 24 hr (right panel) after treatment. Horizontal right bars indicate elevated gene expression, whereas left bars show decreased expression relative to untreated cell lines.

Expression of *HDAC1* after treatment with **(A)** the high concentration (2000 nM) of dasatinib or **(B)** the high concentration (10000 nM) of erlotinib did not satisfy the criteria for concerted expression changes at any time point. **(C)** *SIRT3* expression after treatment with the high concentration of dasatinib, showing concerted upregulation at 24 hr (right panel). **(D)** Expression of *SIRT5* after treatment with the high concentration of erlotinib, with concerted upregulation observed at 24 hr (right panel). **(E)** *HDAC7* expression after treatment with the high concentration of dasatinib showed concerted downregulation at 24 hr (right panel). **(F)** *SIRT2* expression after treatment with the low concentration (100 nM) of dasatinib showed concerted upregulation at 24 hr (right panel).

Colors represent cancer categories. The scale on the bottom represents log_2_ difference between expression values of treated and untreated cell lines. The scale for each microarray experiment is specific to that experiment.

**Figure S4**

**Figure S4 (cont.)**

**Figure S5. Scatterplots of transcriptional changes (log_2_FC) in *HDAC5* expression at 24 hr after treatment with dasatinib vs log(GI50) of the NCI-60 cell lines**

**(A)** High (2,000 nM) concentration of dasatinib. **(B)** Low (100 nM) concentration of dasatinib.

Colors represent cancer categories, as shown in the color legend. Horizontal axis provides log(GI50) values. Vertical axis represents provides log_2_FC values (difference in gene expression between treated and untreated cell lines, provided as log_2_, in treated cells relative to baseline values). **ρ**, Spearman correlation coefficient. ***r****,* Pearson correlation coefficient. ***p*_FDR_**, FDR adjusted *p*-values for Spearman and Pearson correlation analyses.

**Figure S6. Vorinostat-induced changes in normalized expression values of miRNAs with reported regulatory effects on HDAC5, based on the data from the GEO dataset GSE69959**

Shown are the log_2_-transformed normalized changes in expression of the miRNA in the treated vs untreated cell lines values at 6 hr after treatment with 5 μM of vorinostat as compared to the pretreatment levels using publicly available data from GSE69959. The data are for the leukemia cell lines KASUMI1, U937, and K562. Expression changes were averaged among the 5 probes and 3 technical replicates for each miRNA and each cell line. miRNA names in the dataset GSE69959 were, **miR-125a-5p:** hsa-miR-125a-5p; **miR-589-5p:** hsa-miR-589; **miR-217:** hsa-miR-217; **miR-124:** hsa-miR-124; **miR-9:** hsa-miR-9

**Figure S7. Changes in *YAP1* expression in response to treatment with the high concentration of dasatinib and both concentrations of vorinostat**

Shown are transcriptional changes (log_2_FC) of *YAP1* at 2 hr (left panel), 6 hr (middle panel), and 24 hr (right panel) after treatment, as compared to untreated cells. Horizontal right bars indicate elevated gene expression, whereas left bars show decreased expression relative to untreated cell lines. Changes in *YAP1* expression are shown after treatment with **(A)** the high concentration (2000 nM) of dasatinib; **(B)** the low concentration (1000 nM) of vorinostat; and **(C)** the high concentration (5000 nM) of vorinostat. Colors represent cancer categories. Transcriptional downregulation of *YAP1* at 6 and 24 hr satisfied criteria for concerted changes for all three of these conditions. The scale on the bottom represents log_2_ difference between expression values of treated and untreated cell lines. The scale for each microarray experiment is specific to that experiment.

**Figure S7**

**Figure S8. Downregulation of *EPHA2* by dasatinib**

Shown are transcriptional changes (log_2_FC) of *EPHA2* at 2 hr (left panel), 6 hr (middle panel), and 24 hr (right panel) after treatment, as compared to untreated cells. Horizontal right bars indicate elevated gene expression, whereas left bars show decreased expression relative to untreated cell lines. Changes in *EPHA2* mRNA expression are shown after treatment with **(A)** the high (2000 nM) and **(B)** the low (100 nM) concentrations of dasatinib. Transcriptional downregulation of *EPHA2* at each of the three time points satisfied criteria for concerted changes (Supplementary Table S7). The scale on the bottom represents log_2_ difference between expression values of treated and untreated cell lines. The scale for each microarray experiment is specific to that experiment.

**Figure S9. Changes in *MYC* expression in response to treatment with the high concentrations of dasatinib and vorinostat**

Shown are transcriptional changes (log_2_FC) of *MYC* at 2 hr (left panel), 6 hr (middle panel), and 24 hr (right panel) after treatment, as compared to untreated cells. Horizontal right bars indicate elevated gene expression, whereas left bars show decreased expression relative to untreated cell lines. Changes in *MYC* expression are shown after treatment with **(A)** the high concentration (2000 nM) of dasatinib and **(B)** the high concentration (5000 nM) of vorinostat. Colors represent cancer categories. The scale on the bottom represents log_2_ difference between expression values of treated and untreated cell lines. The scale for each microarray experiment is specific to that experiment.
