# Supplementary material for: Association of changes in expression of HDAC and SIRT genes after drug treatment with cancer cell line sensitivity to kinase inhibitors
Source: Epigenetics. 2024 Feb 18;19(1):2309824. doi: 10.1080/15592294.2024.2309824 (PMC10878021; doi:10.1080/15592294.2024.2309824)
Supplement: Supplemental Material [file KEPI_A_2309824_SM1624.zip › Table S3.docx]

**Supplementary Table S3.** Comparison of transcriptional changes in cancer cell lines in response to dasatinib treatment in public NCBI GEO datasets to changes after dasatinib treatment in NCI-TPW

**A. Comparison of changes in mRNA expression in the K-562 cell line in the NCBI GEO dataset GSE51083 to concerted changes in NCI-TPW at 24 hr after treatment with dasatinib**

| **Gene** | **Transcript** | **Source reference ID** | **log_2_FC at 24 hr** | | **Agreement at 24 hr with NCI-TPW 24 hr consensus changes** |
| --- | --- | --- | --- | --- | --- |
| ***HDAC1*** | ILMN_12992 | NM_004964.2 | **0.2137** | No concerted change in NCI-TPW | |
| ***HDAC2*** | ILMN_28766 | NM_001527.2 | **-0.0264** | \|log_2_FC\| ≤ 0.1 | |
| ***HDAC3*** | ILMN_25552 | NM_003883.2 | **0.4350** | No concerted change in NCI-TPW | |
| ***HDAC4*** | ILMN_19556 | NM_006037.3 | **-0.0725** | \|log_2_FC\| ≤ 0.1 | |
| ***HDAC5*** | ILMN_6752 | NM_005474.4 | **-0.0043** | \|log_2_FC\| ≤ 0.1 | |
| ***HDAC6*** | ILMN_7657 | NM_006044.2 | **0.5780** | No concerted change in NCI-TPW | |
| ***HDAC7*** | ILMN_309617 | NM_001098416.2 | **-0.1299** | **Y** | |
| ***HDAC7*** | ILMN_16331 | NM_016596.3 | **-0.2468** | **Y** | |
| ***HDAC7*** | ILMN_307922 | NM_001098415.1 | **0.0338** | \|log_2_FC\| ≤ 0.1 | |
| ***HDAC8*** | ILMN_2001 | NM_018486.1 | **0.0149** | No data in NCI-TPW | |
| ***HDAC9*** | ILMN_11183 | NM_058176.2 | **0.0133** | \|log_2_FC\| ≤ 0.1 | |
| ***HDAC9*** | ILMN_20565 | NM_058177.2 | **0.0088** | \|log_2_FC\| ≤ 0.1 | |
| ***HDAC9*** | ILMN_26338 | NM_014707.1 | **-0.0303** | \|log_2_FC\| ≤ 0.1 | |
| ***HDAC9*** | ILMN_7986 | NM_178425.2 | **0.0115** | \|log_2_FC\| ≤ 0.1 | |
| ***HDAC10*** | ILMN_176841 | NM_032019.4 | **0.1067** | No data in NCI-TPW | |
| ***HDAC11*** | ILMN_26127 | NM_024827.1 | **0.2214** | **Y** | |
| ***SIRT1*** | ILMN_164649 | NM_012238.3 | **0.0817** | \|log_2_FC\| ≤ 0.1 | |
| ***SIRT2*** | ILMN_17601 | NM_030593.1 | **0.2322** | **Y** | |
| ***SIRT2*** | ILMN_21853 | NM_012237.2 | **0.3557** | **Y** | |
| ***SIRT3*** | ILMN_25083 | NM_001017524.1 | **0.0448** | \|log_2_FC\| ≤ 0.1 | |
| ***SIRT3*** | ILMN_25872 | NM_012239.4 | **-0.0634** | \|log_2_FC\| ≤ 0.1 | |
| ***SIRT4*** | ILMN_1274 | NM_012240.1 | **0.1265** | **Y** | |
| ***SIRT5*** | ILMN_18454 | NM_012241.2 | **0.3041** | **Y** | |
| ***SIRT5*** | ILMN_29348 | NM_031244.1 | **0.0343** | \|log_2_FC\| ≤ 0.1 | |
| ***SIRT6*** | ILMN_1560 | NM_016539.1 | **-0.0502** | No concerted change in NCI-TPW | |
| ***SIRT7*** | ILMN_1438 | NM_016538.1 | **-0.0317** | No concerted change in NCI-TPW | |

Shown is the comparison of the direction of transcriptional changes in the K-562 cell line at 24 hr after treatment with 100 nM of dasatinib (after averaging the log_2_FC values among the multiple probes for each transcript and among the three replicate measurements) in GSE51083 [1] to the direction of consensus transcriptional changes in the NCI-TPW dataset at 24 hr after dasatinib treatment. **log_2_FC at 24 hr** indicates the changes in expression in GSE51083; for those changes satisfying|log_2_FC| > 0.1, positive values (upregulation after treatment) are shown in red, and negative values (downregulation after treatment) are shown in blue.

**Y** (highlighted in yellow) indicates an agreement between the direction of transcriptional changes in GSE51083 and the direction of concerted changes in NCI-TPW, for those genes with |log_2_FC| > 0.1 in GSE51083, which also had concerted expression changes at 24 hr after treatment in NCI-TPW, and had the same direction of transcriptional change (both positive or both negative log_2_FC) in both datasets. **|log_2_FC| ≤ 0.1** indicates a small change in the GSE51083 dataset; such genes were excluded from comparisons. The genes with **no concerted change in NCI-TPW** or **no data in NCI-TPW** were also excluded from comparisons.

*The direction of transcriptional changes was the same in GSE51083 and NCI-TPW for each HDAC and SIRT gene satisfying both criteria (|log2FC| > 0.1 in GSE51083 and concerted changes in NCI-TPW).*

For *HDAC7,* concerted expression changes in NCI-TPW at 24 hr were observed only at the high concentration (2000 nM) of dasatinib, and the comparison was made to that condition. For all other genes satisfying concerted expression changes at 24 hr, the comparison of expression changes was made to the low concentration (100 nM) of dasatinib in NCI-TPW, which was the same concentration as that used in GSE51083 experiments.

**B. Comparison of transcriptional changes in NSCLC cell lines in the NCBI GEO dataset GSE69395 at 72 hr after treatment with dasatinib** **to concerted changes in NCI-TPW at 24 hr after treatment with dasatinib**

| **Gene** | **RefSeq transcript ID** | **log_2_FC at 72hr in GSE69395** | | | | **Number of cell lines in agreement with NCI-TPW 24 hr consensus** |
| --- | --- | --- | --- | --- | --- | --- |
|  |  | **A549** | **H661** | **H1666** | **Cal12T** |  |
| ***HDAC1*** | NM_004964 | **-0.1336** | **0.2726** | **-0.2537** | **0.0311** | No concerted change in NCI-TPW |
| ***HDAC2*** | NM_001527 | **-0.1567** | **0.1605** | **-0.0350** | **-0.1530** | **2** |
| ***HDAC3*** | NM_003883 | **-0.0244** | **-0.0780** | **0.0218** | **-0.3490** | No concerted change in NCI-TPW |
| ***HDAC4*** | NM_006037 | **-0.0322** | **0.0054** | **-0.0146** | **-0.0863** | \|log_2_FC\| ≤ 0.1 in all cell lines in GSE69395 |
| ***HDAC5*** | NM_001015053 | **0.1390** | **-0.0067** | **0.0527** | **0.2789** | **2** |
| ***HDAC6*** | NM_006044 | **-0.0065** | **-0.0720** | **0.0551** | **-0.2014** | No concerted change in NCI-TPW |
| ***HDAC7*** | NM_001098416 | **0.0461** | **-0.0049** | **-0.1611** | **-0.0510** | **1** |
| ***HDAC8*** | NM_001166418 | **0.0206** | **0.1212** | **-0.0026** | **-0.2978** | No data in NCI-TPW |
| ***HDAC9*** | NM_001204144 | **0.0412** | **0.0028** | **-0.1586** | **-0.0274** | **1** |
| ***HDAC10*** | NM_001159286 | **-0.0343** | **-0.0826** | **0.0296** | **0.0610** | No data in NCI-TPW |
| ***HDAC11*** | NM_001136041 | **0.1550** | **-0.1019** | **-0.1442** | **0.2587** | **2** |
| ***SIRT1*** | NM_001142498 | **0.0027** | **-0.1135** | **-0.2965** | **0.2497** | No concerted change in NCI-TPW |
| ***SIRT2*** | NM_001193286 | **0.1177** | **0.0616** | **0.2253** | **0.4195** | **3** |
| ***SIRT3*** | NM_001017524 | **0.0378** | **-0.0310** | **0.0649** | **0.0236** | No concerted change in NCI-TPW |
| ***SIRT4*** | NM_012240 | **0.0675** | **-0.0584** | **0.2135** | **-0.1933** | **1** |
| ***SIRT5*** | NM_001193267 | **-0.0420** | **-0.0408** | **-0.0134** | **-0.0388** | \|log_2_FC\| ≤ 0.1 in all cell lines in GSE69395 |
| ***SIRT6*** | NM_001193285 | **0.0769** | **0.0668** | **-0.0714** | **-0.1456** | No concerted change in NCI-TPW |
| ***SIRT7*** | NM_016538 | **-0.2303** | **-0.2149** | **0.0312** | **0.5321** | No concerted change in NCI-TPW |

Shown is the comparison of the direction of transcriptional changes in four non-small cell lung cancer (NSCLC) cell lines at 72 hr after treatment with 150 nM of dasatinib in GSE69395 [2] (after averaging the log_2_FC values among the multiple probes for each transcript) to the direction of consensus transcriptional changes in the NCI-TPW dataset. For changes satisfying |log_2_FC| > 0.1, positive values (upregulation after treatment) are shown in red, and negative values (downregulation) are shown in blue.

**Number of cell lines in agreement with NCI-TPW 24 hr consensus** (highlighted in yellow) shows the number of cell lines in GSE69395 which had the same direction of transcriptional changes with the direction of concerted changes of the consensus in NCI-TPW, for the genes and cell lines with |log_2_FC| > 0.1 in GSE69395 and concerted expression changes at 24 hr after treatment in NCI-TPW. **|log_2_FC| ≤ 0.1 in all cell lines in GSE69395** indicates genes with small transcriptional changes in GSE69395; such genes were excluded from comparisons. The genes with **no concerted change in NCI-TPW** or **no data in NCI-TPW** were also excluded from comparisons.

**References for Table S3**

1. Asmussen J, Lasater EA, Tajon C, Oses-Prieto J, Jun YW, Taylor BS et al. MEK-dependent negative feedback underlies BCR-ABL-mediated oncogene addiction. Cancer Discov. 2014;4:200-15.

2. Peng S, Sen B, Mazumdar T, Byers LA, Diao L, Wang J et al. Dasatinib induces DNA damage and activates DNA repair pathways leading to senescence in non-small cell lung cancer cell lines with kinase-inactivating BRAF mutations. Oncotarget. 2016;7:565-79.
