# Supplementary material for: Association of changes in expression of HDAC and SIRT genes after drug treatment with cancer cell line sensitivity to kinase inhibitors
Source: Epigenetics. 2024 Feb 18;19(1):2309824. doi: 10.1080/15592294.2024.2309824 (PMC10878021; doi:10.1080/15592294.2024.2309824)
Supplement: Supplemental Material [file KEPI_A_2309824_SM1624.zip › Table S4.docx]

**Supplementary Table S4.** Comparison of transcriptional changes in cancer cell lines in response to vorinostat treatment in public NCBI GEO datasets to changes after vorinostat treatment in NCI-TPW

**A. Comparison of changes in mRNA expression of non-transformed and transformed fibroblasts in the NCBI GEO dataset GSE43010** **to concerted changes in NCI-TPW at 24 hr after treatment with vorinostat**

| **Gene** | **RefSeq transcript** | **log_2_FC** | | **Agreement with NCI-TPW consensus** | |
| --- | --- | --- | --- | --- | --- |
|  |  | **BJ** | **BJ LTSTERas** | **BJ** | **BJ LTSTERas** |
| ***HDAC1*** | NM_004964 | **0.7416** | **1.0666** | **Y** | **Y** |
| ***HDAC2*** | NM_001527 | **0.2785** | **0.5441** | No concerted change in NCI-TPW | No concerted change in NCI-TPW |
| ***HDAC3*** | NM_003883 | **2.3352** | **1.8378** | **Y** | **Y** |
| ***HDAC4*** | NM_006037 | **0.8859** | **0.4302** | No concerted change | No concerted change |
| ***HDAC5*** | NM_001015053 | **0.3524** | **0.5306** | **Y** | **Y** |
| ***HDAC6*** | NM_006044 | **-0.0142** | **0.3057** | \|log_2_FC\| ≤ 0.1 | N |
| ***HDAC7*** | NM_001098416 | **-0.2908** | **-0.4955** | **Y** | **Y** |
| ***HDAC8*** | NM_001166418 | **0.2719** | **0.0393** | No data in NCI-TPW | No data in NCI-TPW |
| ***HDAC9*** | NM_001204144 | **-0.1630** | **-0.5525** | **Y** | **Y** |
| ***HDAC10*** | NM_001159286 | **0.2165** | **-0.1633** | No data in NCI-TPW | No data in NCI-TPW |
| ***HDAC11*** | NM_001136041 | **0.4130** | **1.1096** | No concerted change in NCI-TPW | No concerted change in NCI-TPW |
| ***SIRT1*** | NM_001142498 | **-1.8112** | **-0.2612** | **Y** | **Y** |
| ***SIRT2*** | NM_001193286 | **0.8820** | **0.5657** | **Y** | **Y** |
| ***SIRT3*** | NM_001017524 | **0.3759** | **0.8063** | **Y** | **Y** |
| ***SIRT4*** | NM_012240 | **1.7062** | **3.2049** | **Y** | **Y** |
| ***SIRT5*** | NM_001193267 | **0.0183** | **0.0294** | \|log_2_FC\| ≤ 0.1 | \|log_2_FC\| ≤ 0.1 |
| ***SIRT6*** | NM_001193285 | **-0.0839** | **0.3707** | No concerted change in NCI-TPW | No concerted change in NCI-TPW |
| ***SIRT7*** | NM_016538 | **1.2385** | **1.2287** | **Y** | **Y** |

Shown is the comparison of the direction of transcriptional changes in NCBI GEO dataset GSE43010 [1] at 24h hr after treatment with 25 μM of vorinostat, to the direction of consensus transcriptional changes in the NCI-TPW dataset at 24 hr after vorinostat treatment (if concerted transcriptional changes were observed at either high or low concentration for that gene). The dataset GSE43010 included data on BJ (normal fibroblasts) and BJ LTSTERas (transformed fibroblasts using encoding the SV40 large T and small t antigens, hTERT and H-RAS). **log_2_FC at 24 hr** indicates the changes in expression in GSE43010, after averaging the log2FC values among the multiple probes for each transcript and among the three biological replicate measurements. For the values satisfying |log_2_FC| > 0.1 in GSE43010, positive log_2_FC values indicating upregulation after treatment are shown in red, and negative values indicating downregulation are shown in blue.

**Y** (highlighted in yellow) indicates an agreement between the direction of transcriptional changes in the GSE43010 dataset and the direction of concerted changes in NCI-TPW, for those genes with |log_2_FC| > 0.1 in GSE43010, which also satisfied the condition of concerted expression changes at 24 hr after treatment in NCI-TPW, and had the same direction of transcriptional change (both positive or both negative log_2_FC) in both datasets. **|log_2_FC| ≤ 0.1** indicates a small change in expression in the GSE43010 dataset; such genes were excluded from comparisons. The genes with **no concerted change in NCI-TPW** or **no data in NCI-TPW** were also excluded from comparisons.

**B. Comparison of transcriptional changes in the MPNST cell line 90-8TL in the NCBI GEO dataset GSE84205** **to concerted changes in NCI-TPW at 24 hr after treatment with vorinostat**

| **Gene** | **Probeset** | **log_2_FC** | **Agreement with NCI-TPW consensus change** |
| --- | --- | --- | --- |
| ***HDAC1*** | 7899774 | **0.1947** | **Y** |
| ***HDAC2*** | 8129045 | **0.3006** | No concerted change in NCI-TPW |
| ***HDAC3*** | 8114691 | **0.0984** | **Y** |
| ***HDAC4*** | 8060030 | **-0.1533** | No concerted change in NCI-TPW |
| ***HDAC5*** | 8015914 | **0.1459** | **Y** |
| ***HDAC6*** | 8167369 | **-0.4830** | **Y** |
| ***HDAC7*** | 7962659 | **-0.4370** | **Y** |
| ***HDAC8*** | 8173531 | **-0.0154** | No data in NCI-TPW |
| ***HDAC9*** | 8131631 | **-0.1792** | **Y** |
| ***HDAC10*** | 8076937 | **-0.0131** | No data in NCI-TPW |
| ***HDAC11*** | 8077958 | **0.0113** | No concerted change in NCI-TPW |
| ***SIRT1*** | 7927814 | **-0.3427** | **Y** |
| ***SIRT2*** | 8036636 | **0.1150** | **Y** |
| ***SIRT3*** | 7945357 | **-0.1585** | N |
| ***SIRT4*** | 7959148 | **-0.1638** | N |
| ***SIRT5*** | 8116956 | **-0.1488** | **Y** |
| ***SIRT6*** | 8032770 | **0.0384** | No concerted change in NCI-TPW |
| ***SIRT7*** | 8019296 | **-0.1776** | N |

Shown is the comparison of the direction of transcriptional changes in the MPNST cell line 90-8TL at 24 hr after treatment with 2 μM of vorinostat in GSE84205 [2] to the direction of consensus transcriptional changes in the NCI-TPW dataset. For the values satisfying |log_2_FC| > 0.1 in GSE84205, positive values (upregulation after treatment) are shown in red, whereas negative values (downregulation) are shown in blue.

**Y** (highlighted in yellow) indicates an agreement between the direction of transcriptional changes in the GSE84205 dataset and the direction of concerted changes in NCI-TPW, for those genes with |log_2_FC| > 0.1 in GSE84205, which also satisfied the condition of concerted expression changes at 24 hr after treatment in NCI-TPW, and had the same direction of transcriptional change (both positive or both negative log_2_FC) in both datasets. **|log_2_FC| ≤ 0.1** indicates a small change in expression in the GSE84205 dataset; such genes were excluded from comparisons. The genes with **no concerted change in NCI-TPW** or **no data in NCI-TPW** were also excluded from comparisons.

**References for Table S4**

1. Bolden JE, Shi W, Jankowski K, Kan CY, Cluse L, Martin BP et al. HDAC inhibitors induce tumor-cell-selective pro-apoptotic transcriptional responses. Cell Death Dis. 2013;4:e519.

2. Malone CF, Emerson C, Ingraham R, Barbosa W, Guerra S, Yoon H et al. mTOR and HDAC Inhibitors Converge on the TXNIP/Thioredoxin Pathway to Cause Catastrophic Oxidative Stress and Regression of RAS-Driven Tumors. Cancer Discovery. 2017;7:1450-63.
