# Supplementary material for: Association of changes in expression of HDAC and SIRT genes after drug treatment with cancer cell line sensitivity to kinase inhibitors
Source: Epigenetics. 2024 Feb 18;19(1):2309824. doi: 10.1080/15592294.2024.2309824 (PMC10878021; doi:10.1080/15592294.2024.2309824)
Supplement: Supplemental Material [file KEPI_A_2309824_SM1624.zip › Table S7.docx]

**Table S7.** Dasatinib target kinase genes which showed concerted downregulation by dasatinib in the NCI-TPW dataset

| **Dasatinib target** | **Concerted changes after treatment** |
| --- | --- |
| ***ABL2*** | H6↓*, H24↓*, L6↓*, L24↓* |
| ***CSK*** | H24↓, L6↓* |
| ***EPHA2*** | H2↓**, H6↓**, H24↓**, L2↓*, L6↓*, L24↓* |
| ***MAP4K5*** | H6↓*, H24↓* |
| ***YES1*** | H24↓, L24↓ |
| ***ZAK*** | H24↓* |

Listed are the genes encoding dasatinib targets with kinase activity which satisfied the criteria for concerted changes after treatment by dasatinib in the NCI-TPW dataset. All of them were downregulated by dasatinib.

None of the other dasatinib kinase target genes analyzed (***ABL1, BCR, BTK, CSF1R, EPHA5, EPHB4, FGR, FRK, FYN, KIT, LCK, LYN, PDGFRA, PDGFRB, MAPK14,*** and ***SRC***) satisfied the criteria for concerted changes after treatment with dasatinib under any condition.

Downregulation (**↓**) is shown for microarray experiments in which nearly all cell lines were downregulated, and no more than 15 cell lines were upregulated. Expression changes are shown for the high (**H**; 2000 nM) or low (**L**; 100 nM) concentrations of dasatinib. The time when the change was observed is also indicated. Concerted changes for multiple conditions are separated by commas.

***** Concerted downregulation as described above and the difference of log_2_ expression values between treated and untreated cells log_2_FC ≤ -1 in some cell lines

** Concerted downregulation and log_2_FC ≤ -2.5 in some cell lines

For example, **H24↓*** for *ZAK* indicates that it had a concerted downregulation at 24 hr after treatment with the high concentration of dasatinib, with log_2_ expression changes in at least some cell lines ≤ -1
